# Integrated analysis of transcriptomic and proteomic profiles identifies divergent regulatory networks for intramuscular fat in Chinese versus Western Pigs

**DOI:** 10.1371/journal.pone.0354756

**Published:** 2026-09-18

**Authors:** Caoyan Xu, Pengcheng Shen, Chang Sheng Jiang, Ahmed H. Ghonaim, Man Ren, Xiaojin Li, Qianqian Hu, Chonglong Wang

**Affiliations:** 1 College of Animal Science, Anhui Science and Technology University, Chuzhou, China; 2 Anhui Province Key Laboratory of Animal Nutritional Regulation and Health, Chuzhou, China; 3 Anhui Engineering Technology Research Center of Pork Quality Control and Enhance, Chuzhou, China; 4 National Key Laboratory of Agricultural Microbiology, College of Veterinary Medicine, Huazhong Agricultural University, Wuhan, China; 5 Desert Research Center, Cairo, Egypt; 6 Institute of Animal Husbandry and Veterinary Medicine, Anhui Academy of Agricultural Sciences, Hefei, China; Jeju National University, KOREA, REPUBLIC OF

## Abstract

Intramuscular fat (IMF) content is a key determinant of pork quality. In this study, the Chinese indigenous Huai pig (a fat-type breed) and the Western Duroc pig (a lean-type breed) were selected to investigate the molecular mechanisms underlying IMF deposition. Proteomic analysis identified 91 differentially expressed proteins (DEPs) between the two breeds. Gene Ontology (GO) and Kyoto Encyclopedia of Genes and Genomes (KEGG) enrichment analyses revealed that several key lipid metabolism-related pathways were significantly enriched, including the Lipid metabolic process, AMPK signaling pathway, nicotinate and nicotinamide metabolism, and Fat digestion and absorption. Western blotting further confirmed the reliability of the proteomic results. Integrated transcriptomic and proteomic analyses highlighted significant enrichment of the PPAR signaling pathway, which plays a central role in lipid metabolism. These findings suggest that enhanced fatty-acid uptake contributes to increased lipid deposition in muscle tissue. Additionally, several potential candidate genes associated with IMF deposition were identified, including *CD36*, *SCARB2*, *ACADVL*, *ACADSB*, *ACADM*, *UNC119B*, *PAS-4*, and *APOA1*. Collectively, this study provides valuable insights into the molecular regulation of IMF deposition in pigs and offers a theoretical basis and genetic resources for improving pork quality and advancing molecular breeding.

## Introduction

Pork is one of the most important sources of animal protein worldwide and a major component of human diets. As living standards continue to improve, consumer demand for high-quality pork has increased. Intramuscular fat (IMF) content is a key determinant of pork quality, exerting a major influence on meatflavor, juiciness, and tenderness [1,2]. The deposition of IMF in pigs is regulated by a complex interplay of genetic, environmental, and nutritional factors [3]. The Duroc pig, a representative Western commercial breed, is characterized by a high lean meat percentage and rapid growth performance. In contrast, the Huai pig, a native Chinese breed, is known for its strong adaptability to coarse feed, high IMF content, and superior meat quality [4,5]. However, the molecular mechanisms responsible for the substantial differences in IMF deposition and meat quality between Chinese indigenous and Western commercial pig breeds remain incompletely understood.

In animals, muscle growth, development, and IMF deposition are ultimately regulated by dynamic changes in protein expression, modification, and function. Therefore, understanding the proteomic landscape is critical for elucidating the biological basis of pork quality. The integration oftranscriptomic and proteomic analyses provides a powerful approach to comprehensively explore the molecular networks underlying muscle development and lipid metabolism in pigs. Advances in high-throughput sequencing and mass spectrometry technologies have facilitated the widespread application of these omics tools in animal genetics and meat science research. For example, Wei et al. [6] reported a substantial overlap between differential- ly expressed genes (DEGs) and differentially expressed proteins (DEPs), suggesting that these genes play key roles in regulating skeletal muscle fiber formation. Similarly, Shang et al. [7] integrated transcriptomic and proteomic data from Yorkshire, Wujin, and Tibetan pigs, identified 228 genes showing differential expression at both the mRNA and protein levels, among which 209 exhibited consistent expression patterns. However, the overall correlation between mRNA and protein expression levels was relatively low, suggesting a potential role of post-transcriptional and translational regulation in shaping protein abundance. Consequently, proteomic profiling is essential for achieving a complete understanding of the molecular mechanisms controlling porcine muscle growth and IMF deposition.

In the presentstudy, we performed integrated proteomic and transcriptomic analysesof longissimus dorsi (LD) muscle tissues from Huai and Duroc pigs. We identified key DEGs and DEPs potentially involved in the regulation of IMF deposition and muscle development. These findings provide valuable insights into the molecular mechanisms underlying the distinct differences in IMF content between Chinese and Western pig breeds, offering a theoretical foundation for the genetic improvement of pork quality.

## Materials and methods

### Ethics statement and animal sample collection

The study was approved by the Anhui University of Science and Technology Subcommittee of Experimental Animal Ethics with license number of 2024−016. Also, all the experiments in the manuscript follows the recommendations in the Animal Research Reporting in Vivo Experiments (ARRIVE) guidelines.The study was conducted in accordance with relevant guidelines and regulations.

Adult Huai pigs (n = 3) and Duroc pigs (n = 3) were randomly selected as experimental subjects. The six castrated male pigs used in this experiment were provided by the Anhui Haoxiang Agriculture and Animal Husbandry Co., Ltd., Bozhou, China. All male piglets were surgically castrated at **7** days of life. Throughout the experimental period, all animals were housed under identical conditions with *ad libitum* access to water and a commercial corn-soybean meal diet formulated to meet the National Research Council (NRC, 2012) nutrient requirements. At exactly 180 days of age, following a 12 h fast, the average live body weight was recorded as 86.3 ± 4.0 kg for Huai pigs and 113.6 ± 4.9 kg for Duroc pigs. The experimental pigs were then slaughtered at the commercial abattoir of Anhui Haoxiang Agriculture and Animal Husbandry Co., Ltd. (Bozhou, China). To minimize animal suffering, the pigs were rendered unconscious using electrical stunning (1.5 A, 5 s) and immediately euthanized via exsanguination by severing the carotid arteries and jugular veins. Death was confirmed by the cessation of heartbeat and respiration. A section of the LD muscle near the third or fourth rib was dissected immediately after euthanasia. Each sample was rapidly frozen in liquid nitrogen and transferred to a −80°Cfreezer for long-term storage. Samples from Huai pigs were labeled HB1, HB2, and HB3, while those from Duroc pigs were labeled DB1, DB2, and DB3.

### Protein extraction and enzymatic hydrolysis

LD muscle samples were ground in liquid nitrogen to a fine powder. Each sample was homogenized in 200 μL of lysis buffer (8 M urea, 1% SDS) and sonicated on ice. The lysate was centrifuged at 16,000 × g for 30 minutes at 8 °C, and the resulting supernatant containing soluble proteins was collected. Protein concentration was determined using a bicinchoninic acid (BCA) Protein Assay Kit (Thermo Scientific, USA), following the manufacturer’s instructions. The extraction efficiency was evaluated by SDS-PAGE.

An aliquot of the protein extract was denatured in 100 μL of lysis buffer (8 M urea, 100 mM TEAB, pH 8.5). The proteins were digested with trypsin in 100 mM TEAB buffer at 37°C for 4 hours, followed by the addition of trypsin and CaCl_2_ for overnight incubation to ensure complete digestion. The reaction was terminated by acidifying the mixtureto pH < 3 with formic acid. Samples were centrifuged at 12,000 × g for 5 minutes at room temperature to remove insoluble debris.

The resulting supernatant was desalted using a C18 column pre-equilibrated with wash buffer (0.1% formic acid, 3% acetonitrile). After washing, peptides were eluted with elution buffer (0.1% formic acid, 70% acetonitrile) and lyophilized to dryness for subsequent LC-MS/MS analysis.

### LC-MS/MS analysis

LC-MS/MS analysis was performed using a Vanquish Neo UHPLC system (Thermo Fisher Scientific, USA) coupled to an Orbitrap Astral mass spectrometer (Thermo Fisher Scientific) operated in data-independent acquisition (DIA) mode. The analysis was carried out at Shanghai Majorbio Bio-Pharm Technology Co., Ltd. (Shanghai, China).

Raw DIA data were processed using Spectronaut software (Version 19). Peptide spectrum matches (PSMs) were filtered with a ≥ 99% confidence cut-off, and proteins supported by at least one unique peptide were retained. The false discovery rate (FDR) was controlled at < 1% at both the peptide and protein levels.

### Identification and functional enrichment analysis of differentially expressed proteins (DEPs)

Proteins with a fold change ≥ 1.5 or ≤ 0.67 and an adjusted *p*-value (FDR) < 0.05 were considered using the Benjamini-Hochberg method for multiple testing correction, were considered DEPs. Functional annotation included analyses of subcellular localization, Gene Ontology (GO) enrichment, and Kyoto Encyclopedia of Genes and Genomes (KEGG) pathway enrichment [8]. GO terms and KEGG pathways with a Benjamini-Hochberg adjusted *p*-value < 0.05 were regarded as significantly enriched.

### Protein-protein interaction (PPI) network construction

A Protein-Protein Interaction (PPI) network was constructed using the STRING database (v11.5) based on identified DEPs and visualized using Cytoscape software (v3.7.1). To identify hub proteins, the CytoHubba plugin was employed to calculate topological parameters including degree and betweenness centrality. This analysis facilitated the identification of highly interconnected modules and core regulatory proteins potentially associated with meat quality traits in Huai and Duroc pigs.

### Western blot validation

Proteins were extracted from LD muscle tissues using RIPA lysis buffer (Thermo Scientific), and concentrations were determined using a BCA assay. Equal amounts of protein were separated by SDS-PAGE and transferred onto PVDF membranes. After blocking, membranes were sequentially incubated with primary antibodies and appropriate HRP-conjugated secondary antibodies, followed by signal development. Band intensities were quantified using Image J 1.39U software to compare relative expression levels between Huai and Duroc pigs.

### Integrated transcriptome and proteome analysis

To explore the regulatory relationships between mRNA and protein expression, integrated transcriptomic and proteomic analyses were performed [9]. Correlation analyses were conducted between DEGs and DEPs, as well as their enriched GO terms and KEGG pathways. *p* < 0.05 was considered statistically significant.

### Statistical analysis

All quantitative data are expressed as mean ± standard deviation (SD). Statistical significance between groups was assessed using a two-tailed Student’s t-test. Values of *p* < 0.05 were considered statistically significant, and *p* < 0.01 denoted a highly significant difference. Each experiment was performed in triplicate, and figures were generated using using GraphPad Prism 9.

## Results

### Data quality control

**Fig 1:**
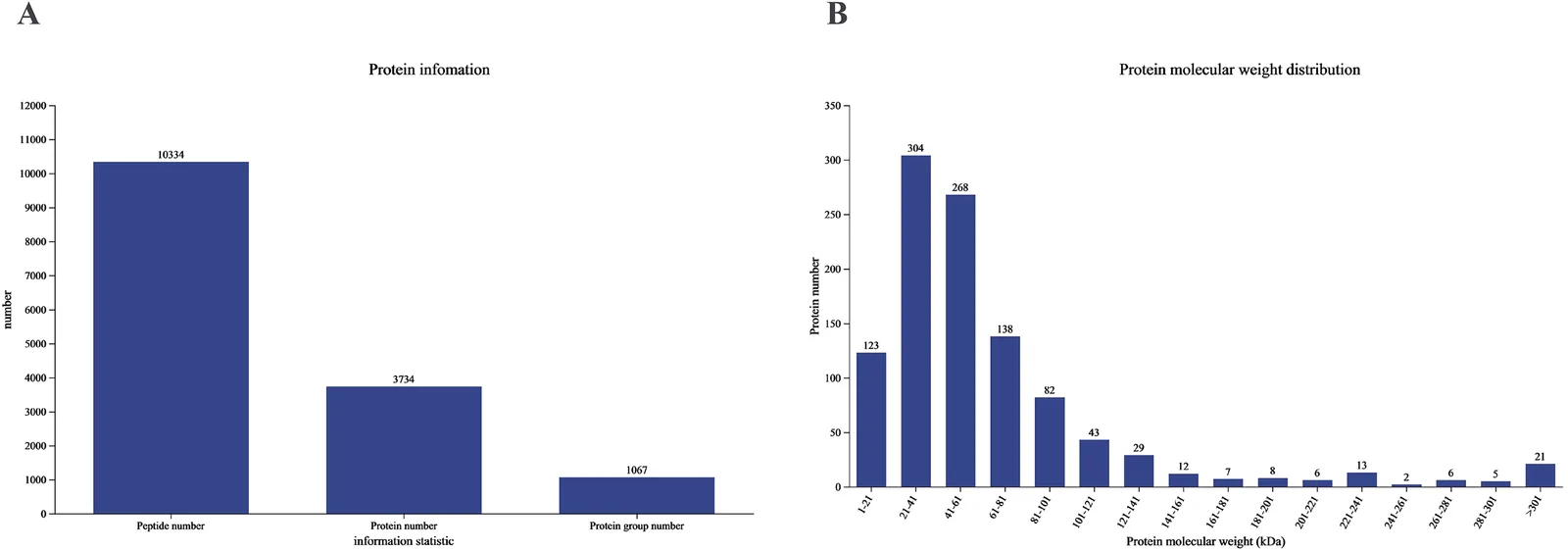
Characterization of proteins from Huai (HB)and Duroc (DLB)LD muscle samples. (A) Overview of identified proteins. (B) Distribution of the molecular weights.

**Fig 2:**
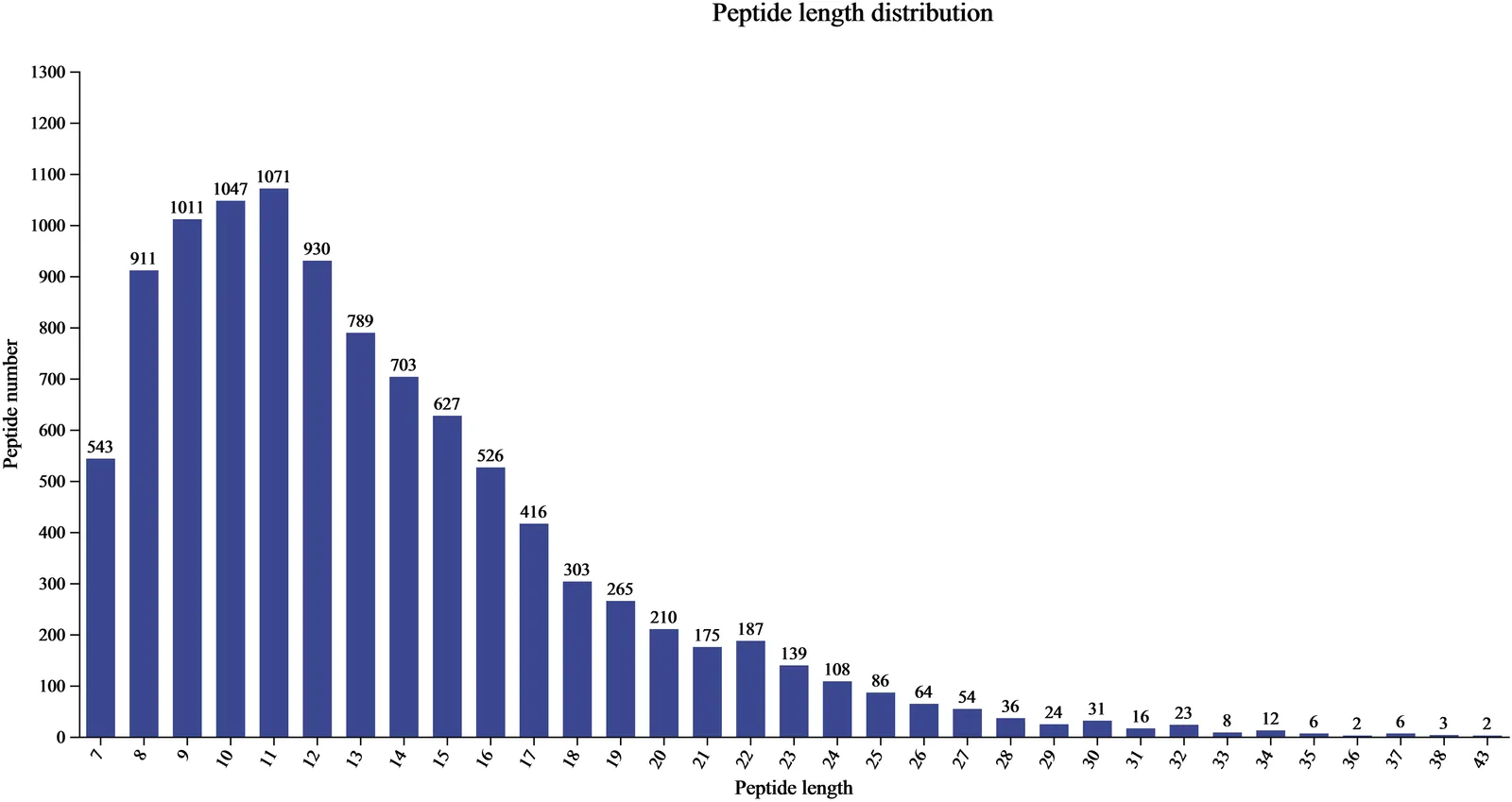
Length distribution of identified peptides.

**Fig 3:**
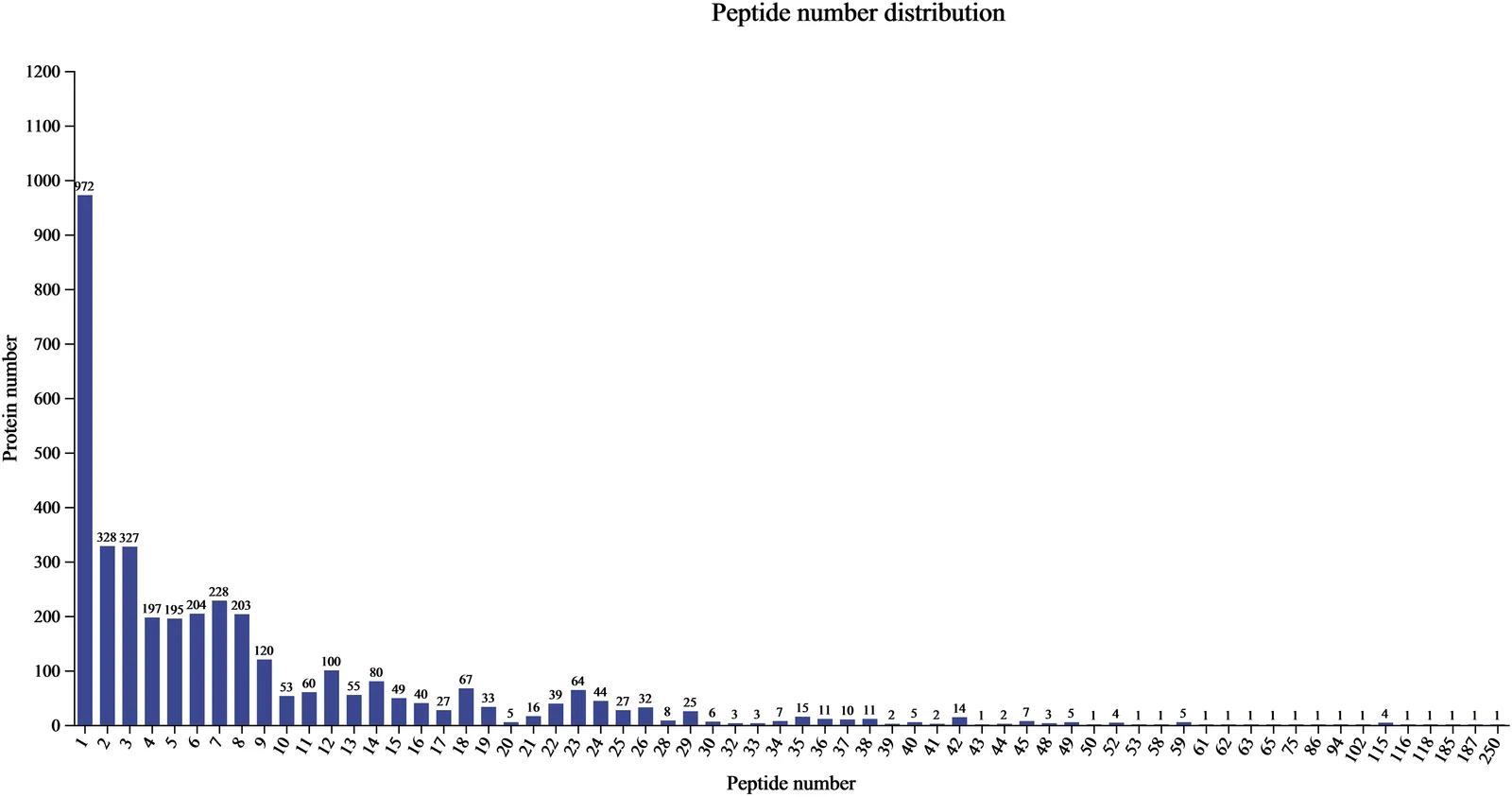
Distribution of proteins with varying peptide numbers.

**Fig 4:**
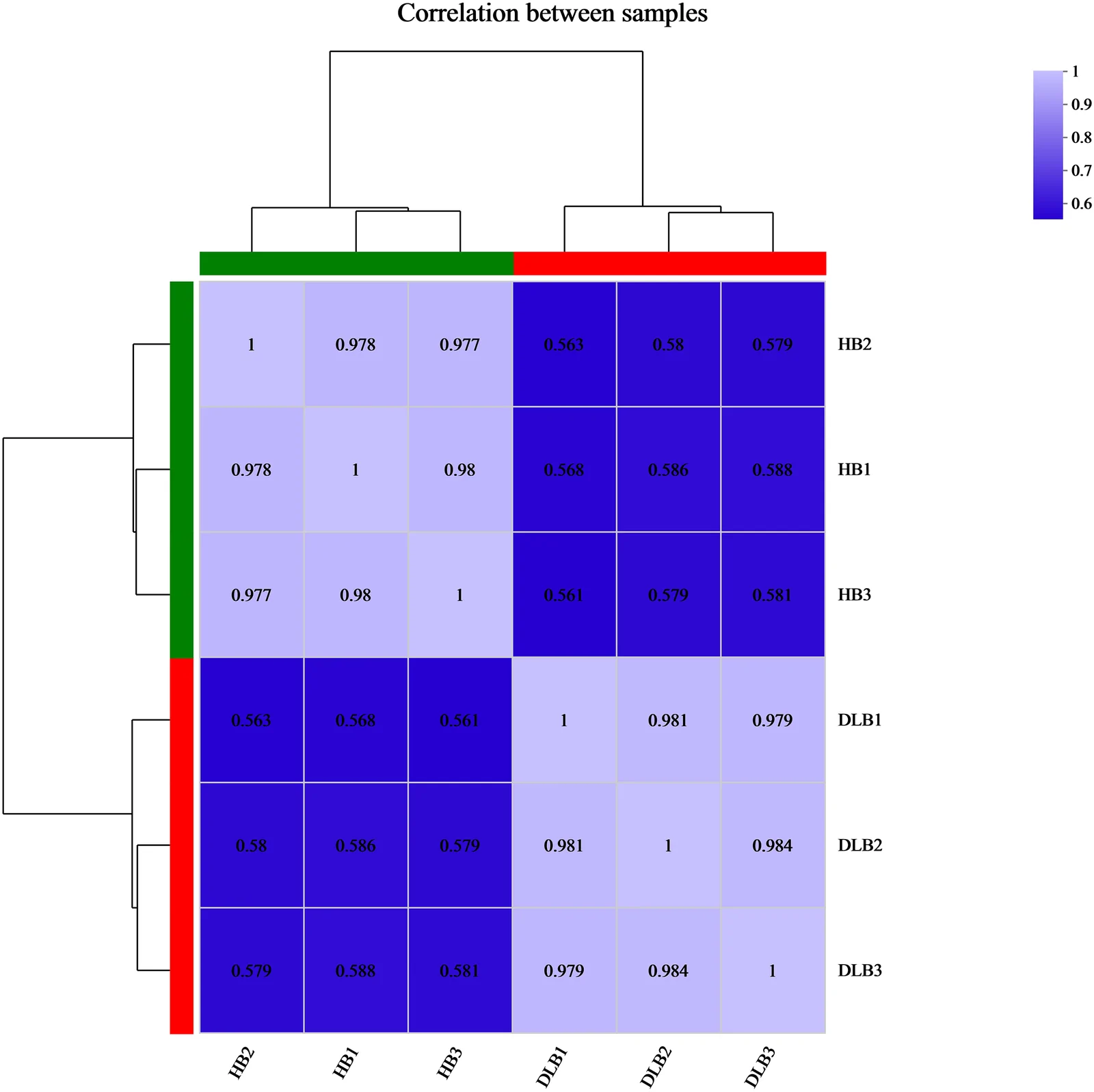
Heatmap showing sample correlations among biological replicates.

LD muscle tissues from Huai and Duroc pigs were analyzed using data-independent acquisition (DIA) mass spectrometry. A total of 10,334 peptides, 1,067 protein groups, and 3,734 proteins were identified (Fig 1a). The molecular weights of the identified proteins exhibited a broad distribution, with most ranging between 21–61 kDa (Fig 1b). The length distribution of identified peptides is shownin (Fig 2). As shown in (Fig 3), most proteins were identified by 1–8 peptides, with, 972 proteins identified based on a single peptide. Biological replicates between the two breedsdemonstrated high reproducibility, with Pearson’s R^2^ > 0.99 within groups and > 0.80 across differential protein libraries (Fig 4). These results confirm that the proteomic data are of high quality and suitable for subsequent analyses.

### Identification of differentially expressed proteins (DEPs)

**Fig 5:**
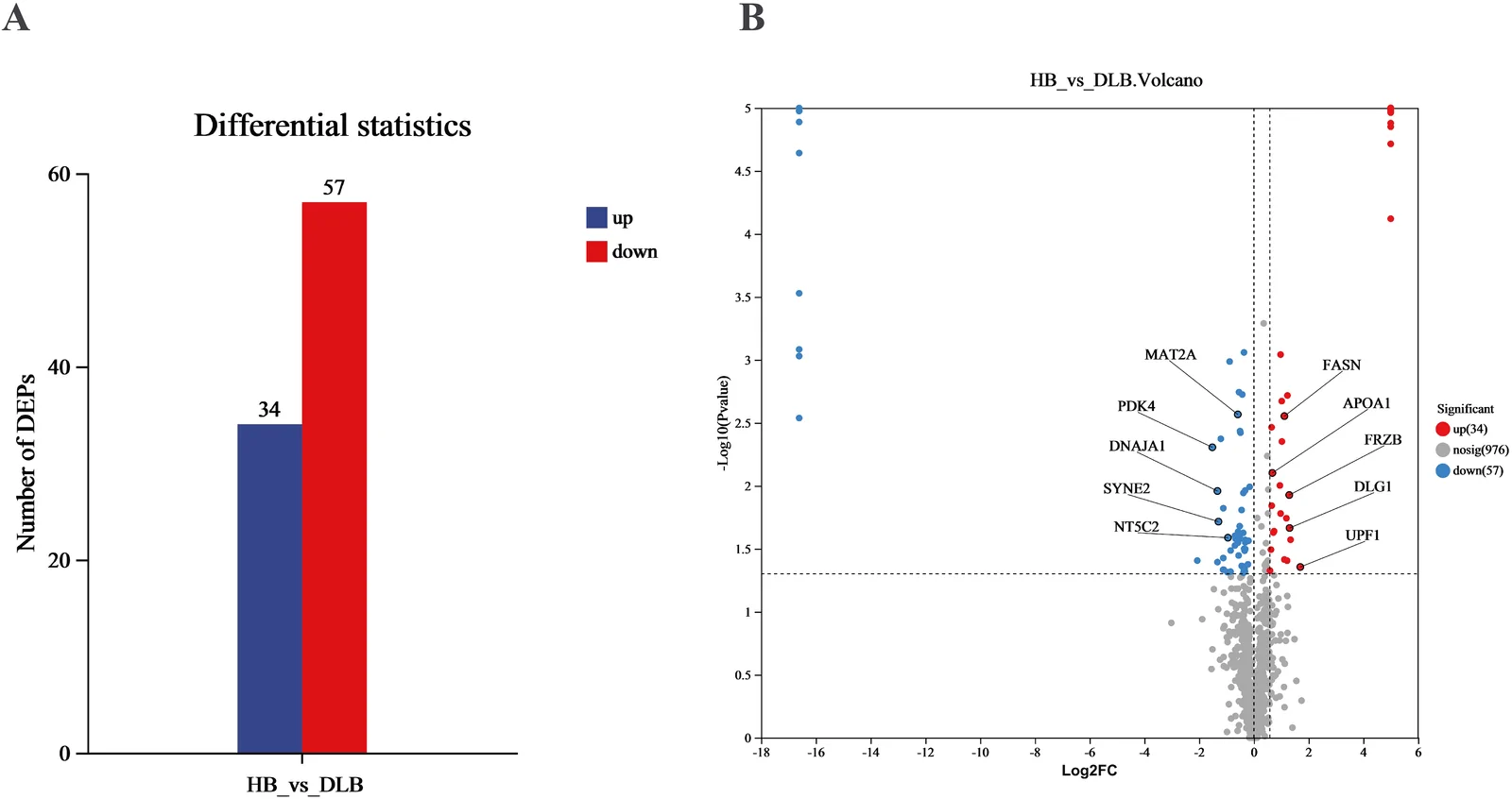
Differentially expressed proteins (DEPs) between HB and DLB groups. (A) Volcano plot of DEPs. (B) Bar chart plot showing numbers of up- and down-regulated proteins.

**Fig 6:**
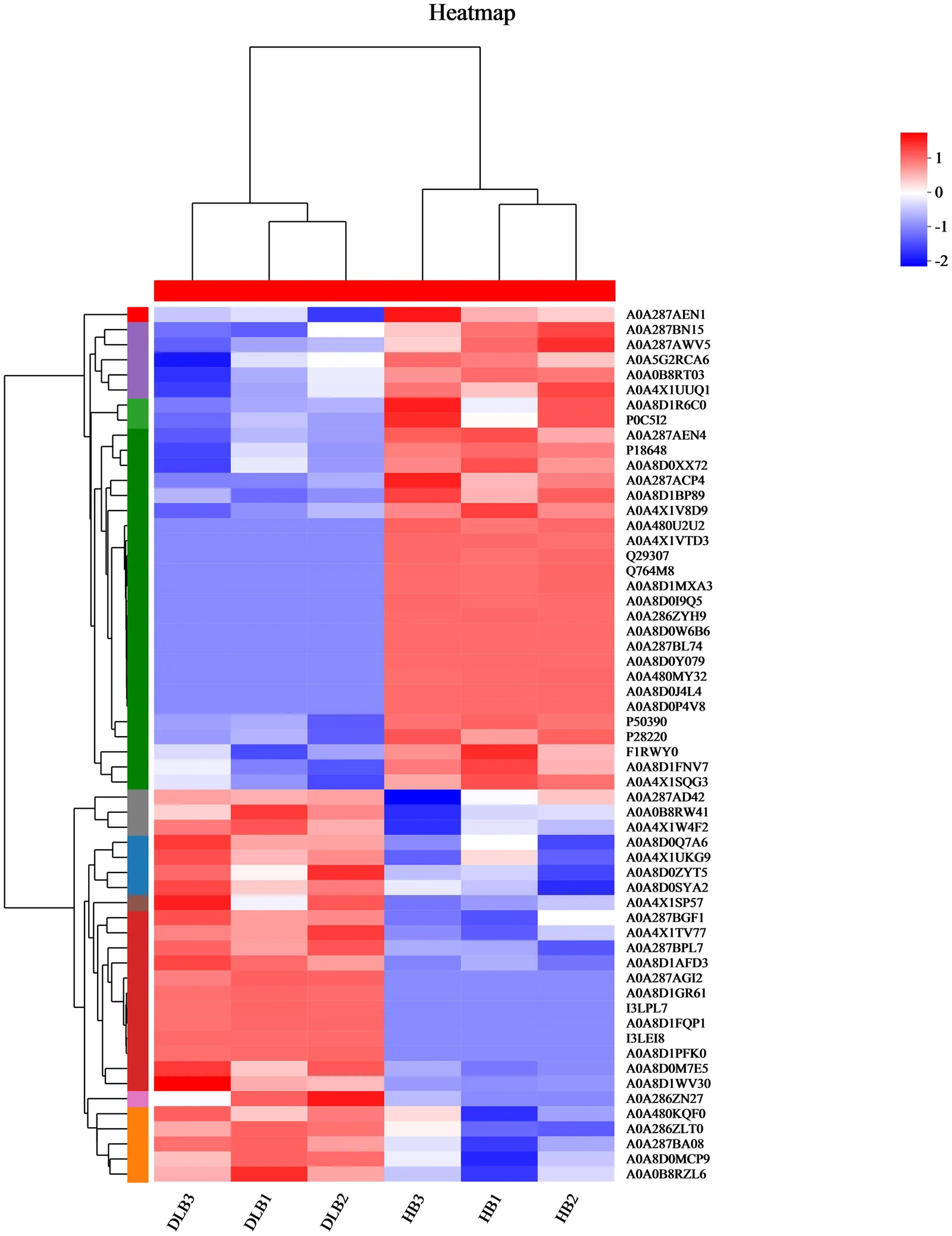
Hierarchical clustering heatmap of DEPs between HB and DLB groups.

**Fig 7:**
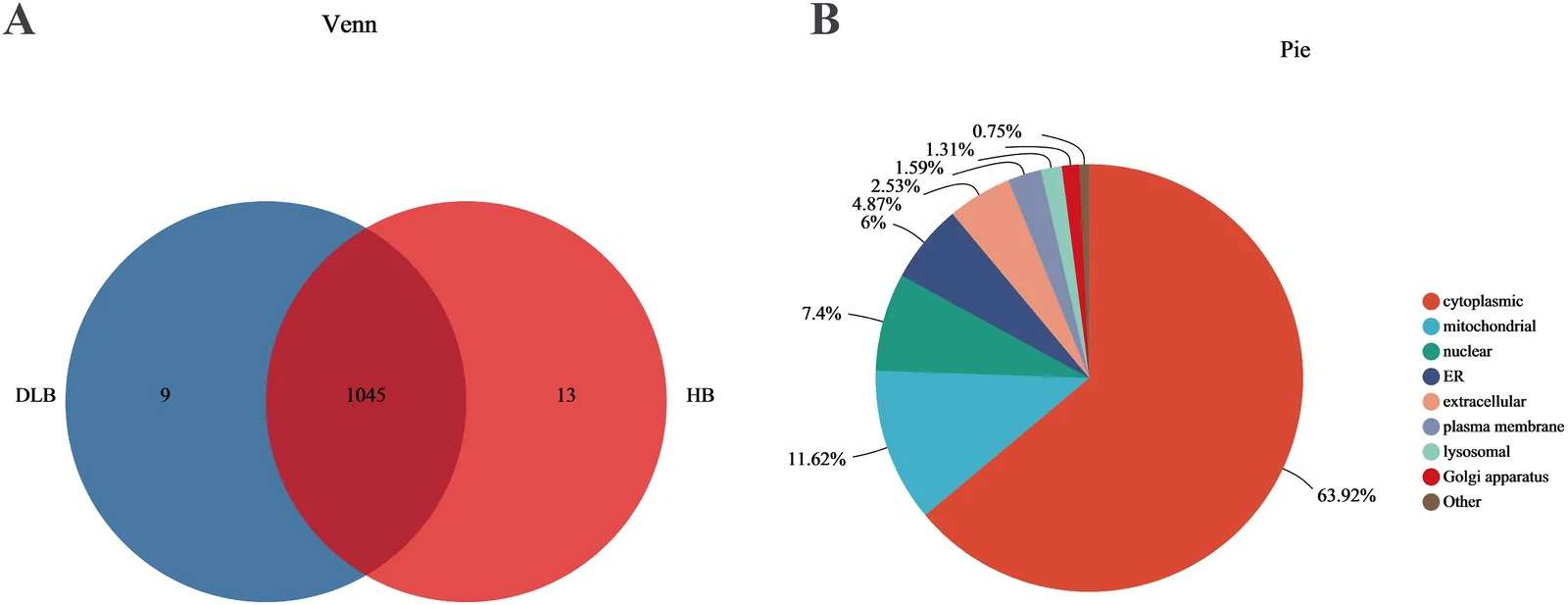
(A) Venn diagram showing overlapping proteins (B) Subcellular localization distributionof DEPs.

Differential expression analysis of LD muscle tissues from Huai and Duroc pigs identified 91 DEPs, including34 up-regulated and 57 down-regulated proteins in Huai pigs relative to Duroc pigs (Fig 5a,b). Hierarchical clustering of DEPs revealed clear separation between the two breeds, indicating distinct proteomic signatures (Fig 6). A total of 1,045 proteins were commonly quantified in the LD muscle of both breeds (Fig 7a). Subcellular localization analysis showed that DEPs were mainly distributedin the cytoplasm (63.92%), mitochondria (11.62%), nucleus (7.40%), and extracellular space (6.00%) (Fig 7b).

### Functional enrichment and bioinformatics analyses

**Fig 8:**
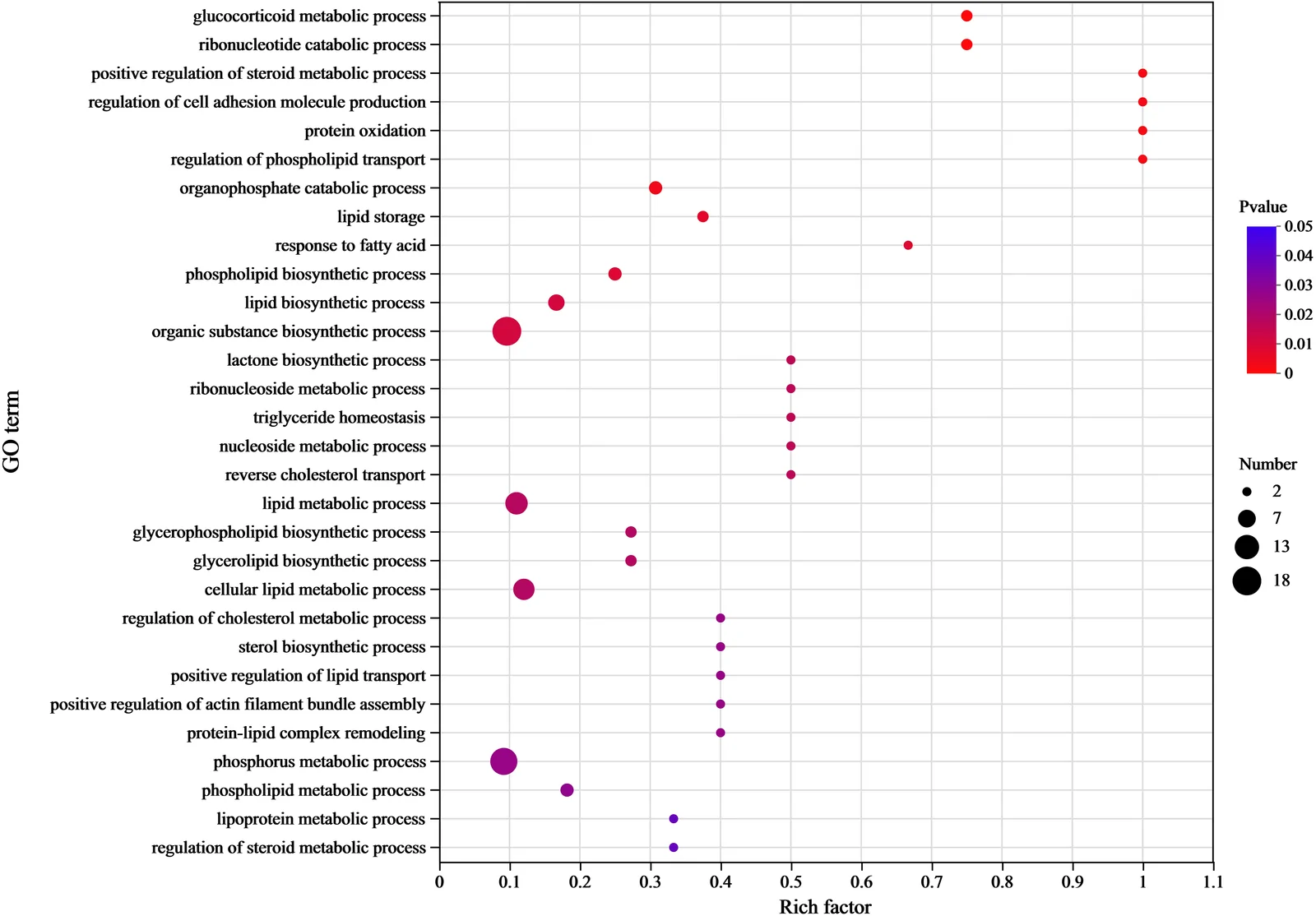
Bubble plot of GO enrichment analysis for DEPs between HB vs. DLB groups.

To elucidatethe biological rolesof DEPs, GO enrichment analysis was performed. In total, 190 GO terms were significantly enriched (*p* < 0.05), comprising 155 terms (81.5%) in the biological process (BP) category, 6 (3.2%) in cellular component (CC), and 29 (15.3%) in molecular function (MF) (Fig 8).

The most significantly enriched BP terms included organic substance biosynthetic process, lipid biosynthetic process, and lipid metabolic process. Significantly enriched CC terms included apical plasma membrane, bicellular tight junction, and spherical high-density lipoprotein particle. Enriched MF terms included lipoprotein particle receptor binding, apolipoprotein A-I receptor binding, and heat shock protein binding. These results suggest that the DEPs are predominantlyinvolved in lipid metabolism and biosynthesis, indicating their key roles in intramuscular fat deposition in both Huai and Duroc pigs.

**Fig 9:**
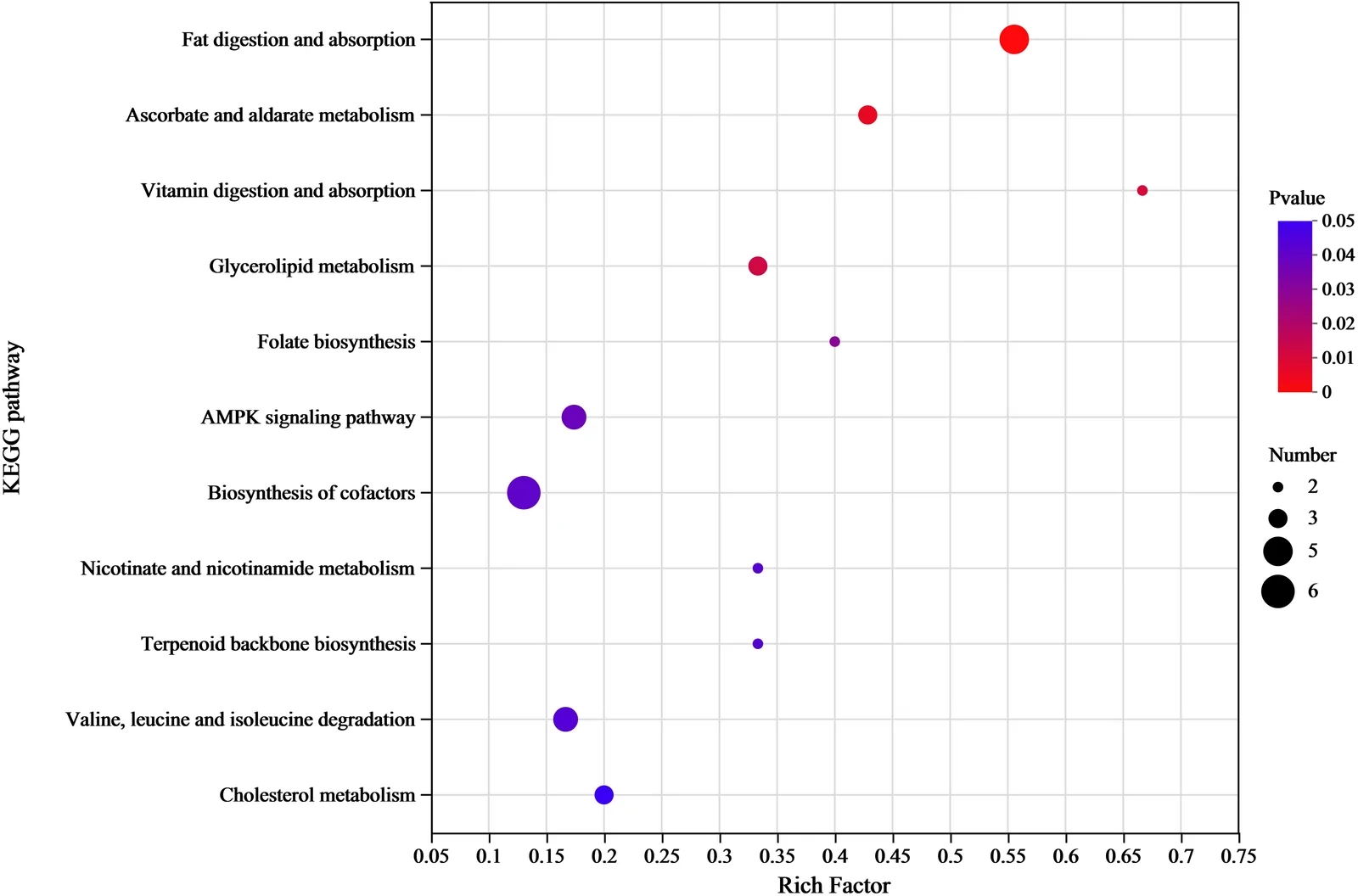
Bubble plot of KEGG enrichment analysis for DEPs between HB vs. DLB groups.

KEGG pathway enrichment analysis assigned the DEPs to 167 pathways, of which 11 were significantly enriched (*p* < 0.05) (Fig 9). Notably, the AMPK signaling pathway, cholesterol metabolism, and fat digestion and absorption pathways were among the most significantly enriched, further underscoring their involvement in lipid metabolic regulation.

### Protein-protein interaction (PPI) network analysis

**Fig 10:**
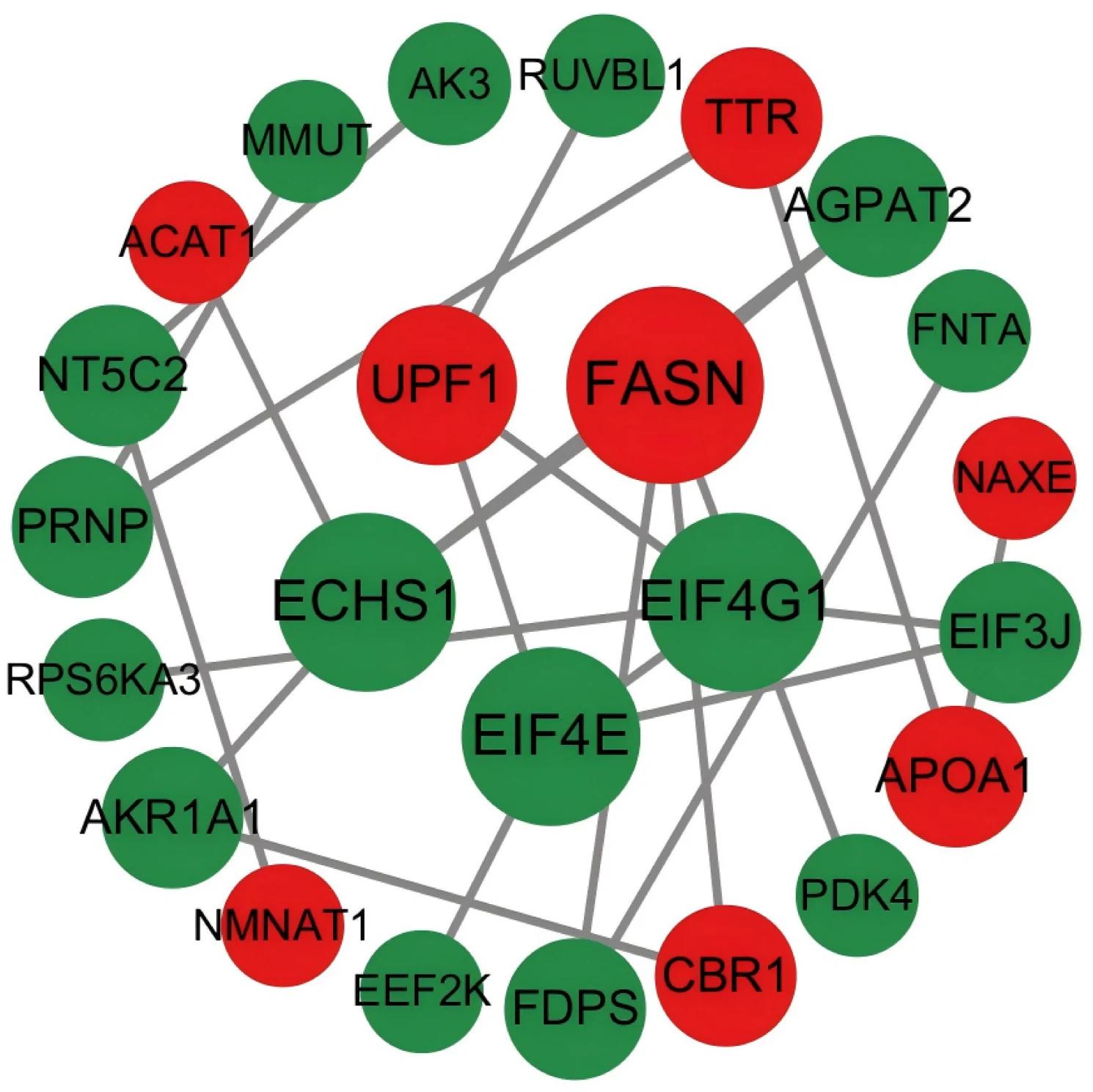
PPI network of DEPs between HB and DLB groups. Red nodes represent up-regulated proteins and green nodes represent down-regulated proteins. Node size corresponds to connectivity (degree).

A PPI network was constructed using the STRING database and visualized with Cytoscape v3.2.1. The network revealed extensive protein-protein interactions among the DEPs, forming several functional modules (Fig 10 and Table S1 in S1 File). These modules were primarily enriched in the AMPK signaling pathway, fat digestion and absorption, and cholesterol metabolism.

The PPI network comprised 8 up-regulated and 16 down-regulated proteins. Hub nodes, including FASN, EIF4G1, EIF4E, ECHS1, and UPF1, exhibited high connectivity and are likely key regulators of lipid metabolism and IMF deposition in pigs.

### Validation of differentially expressed proteins by Western blotting

**Fig 11:**
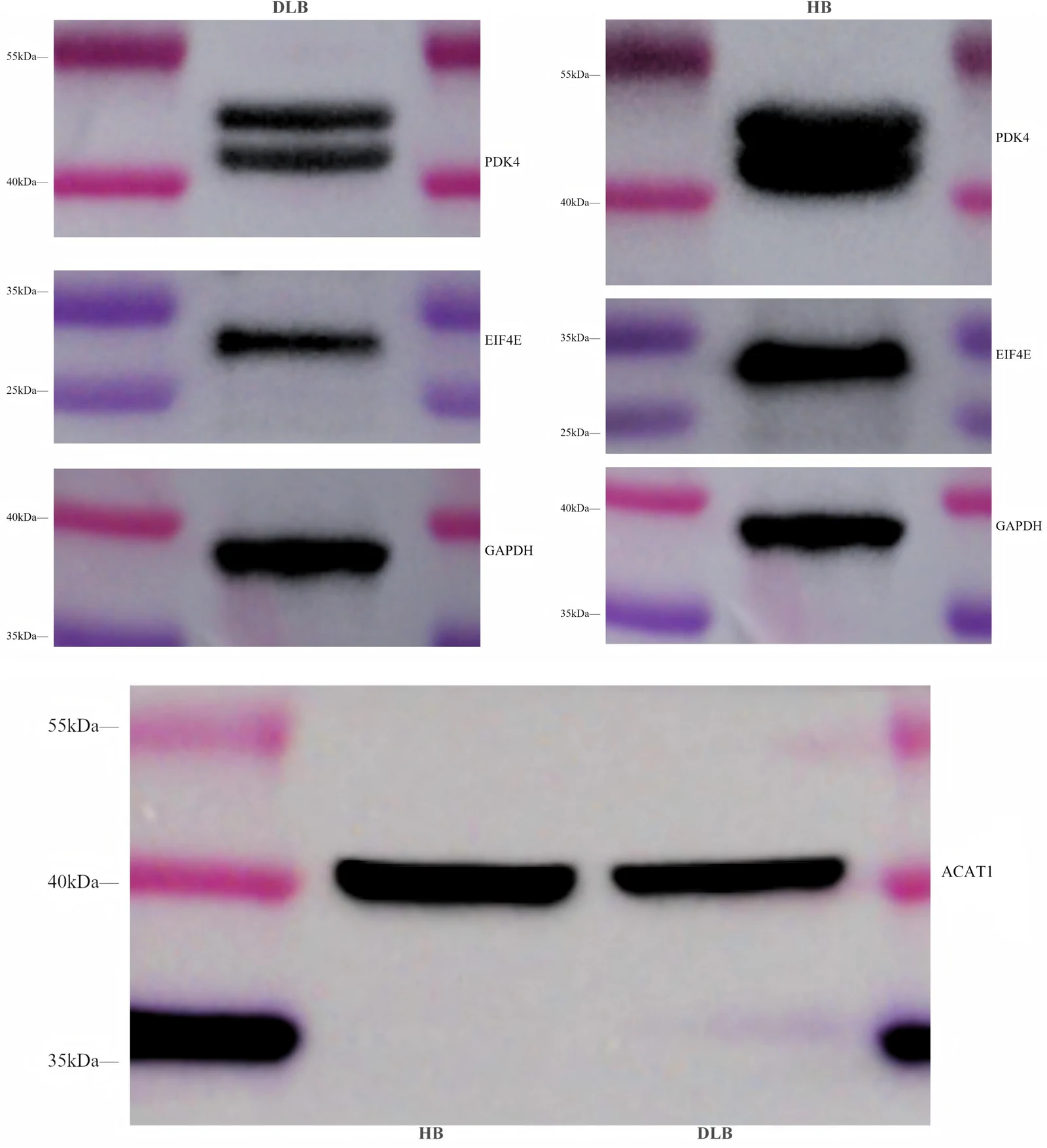
Western-blot results for ACAT1, PDK4, and EIF4E proteins in LD tissues from Huai and Duroc pigs.

**Fig 12:**
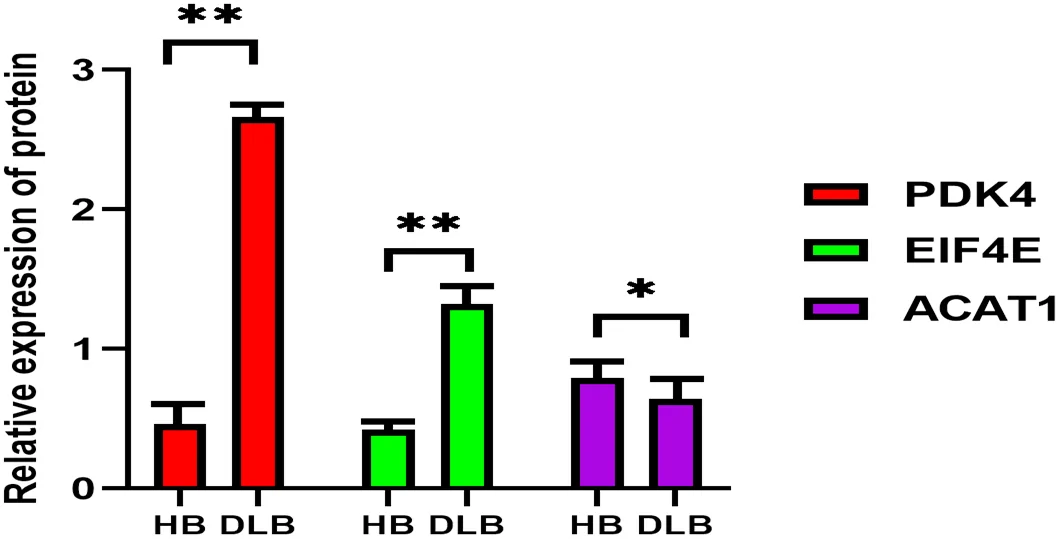
Relative expression levels of ACAT1, PDK4, and EIF4E in LD tissues from Huai and Duroc pigs.

**Fig 13:**
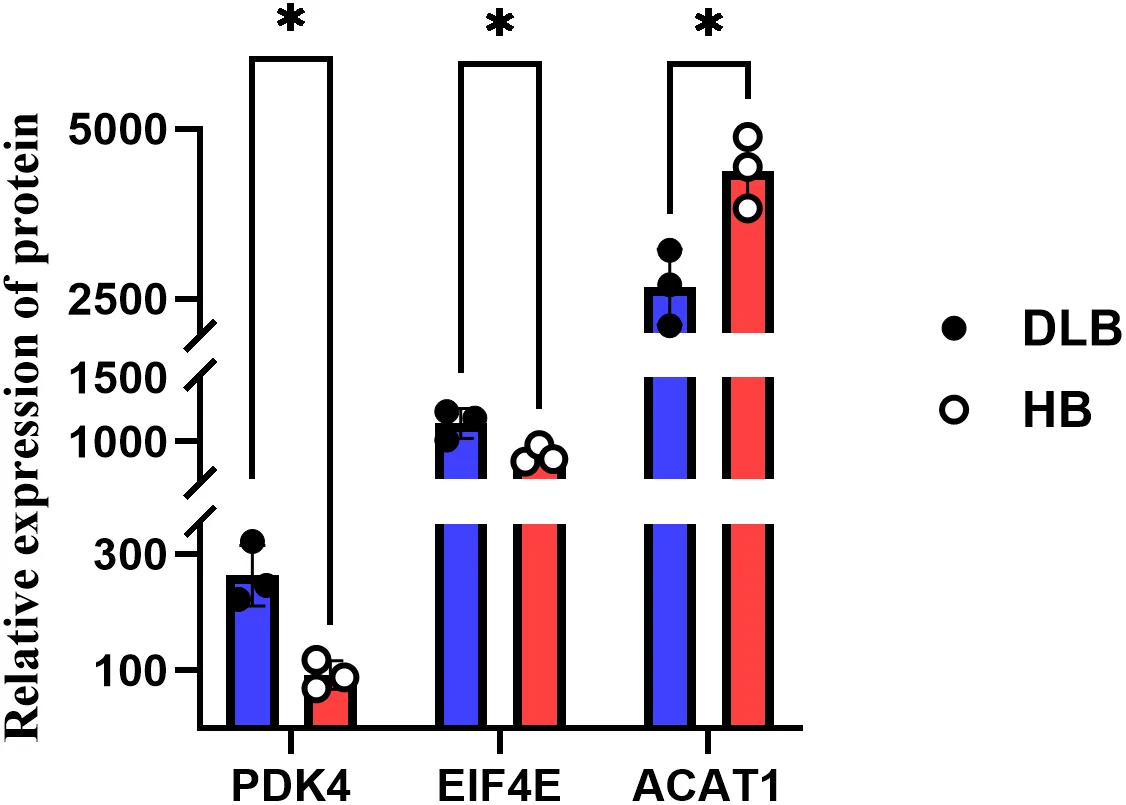
Expression profiles of PDK4, EIF4E, and ACAT1 based on proteomic analysis in Huai and Duroc pigs.

To validate the proteomic results, three DEPs, ACAT1, PDK4, and EIF4E, were randomly selected for Western blot analysis. As shown in (Fig 11), the expression of ACAT1 was significantly higher in Huai pigs, whereas PDK4 and EIF4E were significantly lower compared with Duroc pigs (*p* < 0.05). Quantitative analysis of gray values (Fig 12) confirmed that the expression patterns were consistent with the DIA proteomic data, supporting the reliability of the proteomic findings. Furthermore, we explicitly displayed the individual-level protein abundances of these key targets obtained from the DIA sequencing in Fig 13. This robust cross-validation strongly supports the reliability of our proteomic findings.

### Integrative transcriptomic and proteomic analysis

**Fig 14:**
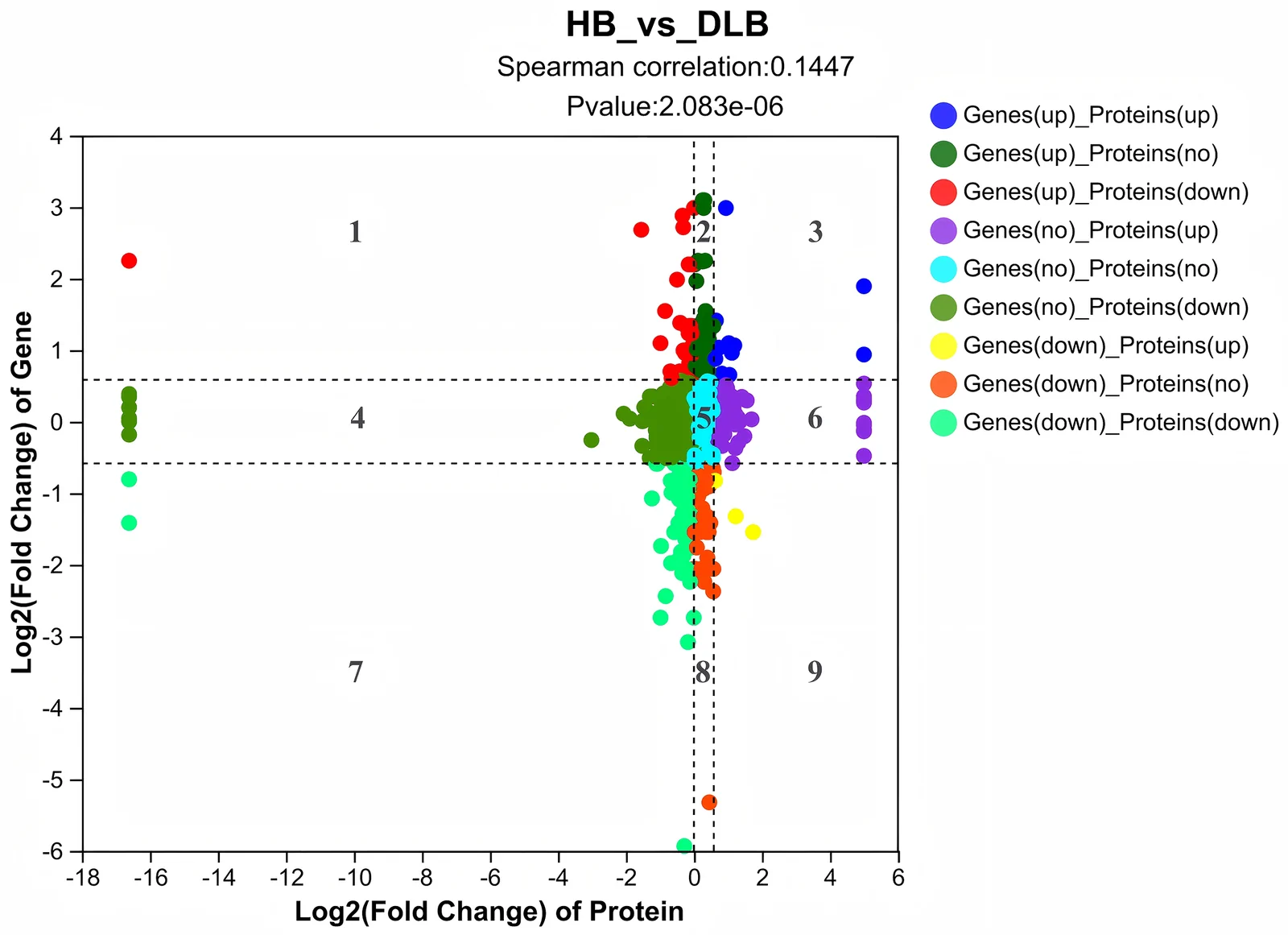
Nine-quadrant correlation plot of DEGs and DEPs between HB and DLB groups.

To further explore the molecular mechanisms underlying LD muscle development and IMF deposition, correlation analyses were conducted between the transcriptomic and proteomic datasets. Given the overall low concordance between mRNA and protein levels, we stratified all DEGs and DEPs into concordant and discordant candidates using a nine-quadrant plot (Fig 14) to better dissect the potential post-transcriptional regulations.

The overall correlation between mRNA and protein expression was modest (Pearson’s r = 0.1447, *p* < 0.05). This low correlation suggests the potential involvement of extensive post-transcriptional or post-translational modifications. However, this discrepancy may also be partially attributed to technical disparities between omics platforms and the inherent temporal dynamics between mRNA and protein half-lives. In total, 576 genes exhibited concordant expression trends at both transcriptional and protein levels, including 12 genes up-regulated and 564 genes down-regulated in both datasets. Conversely, 49 genes were up-regulated at the transcript level but down-regulated at the protein level, while 53 genes showed the opposite pattern.

**Fig 15:**
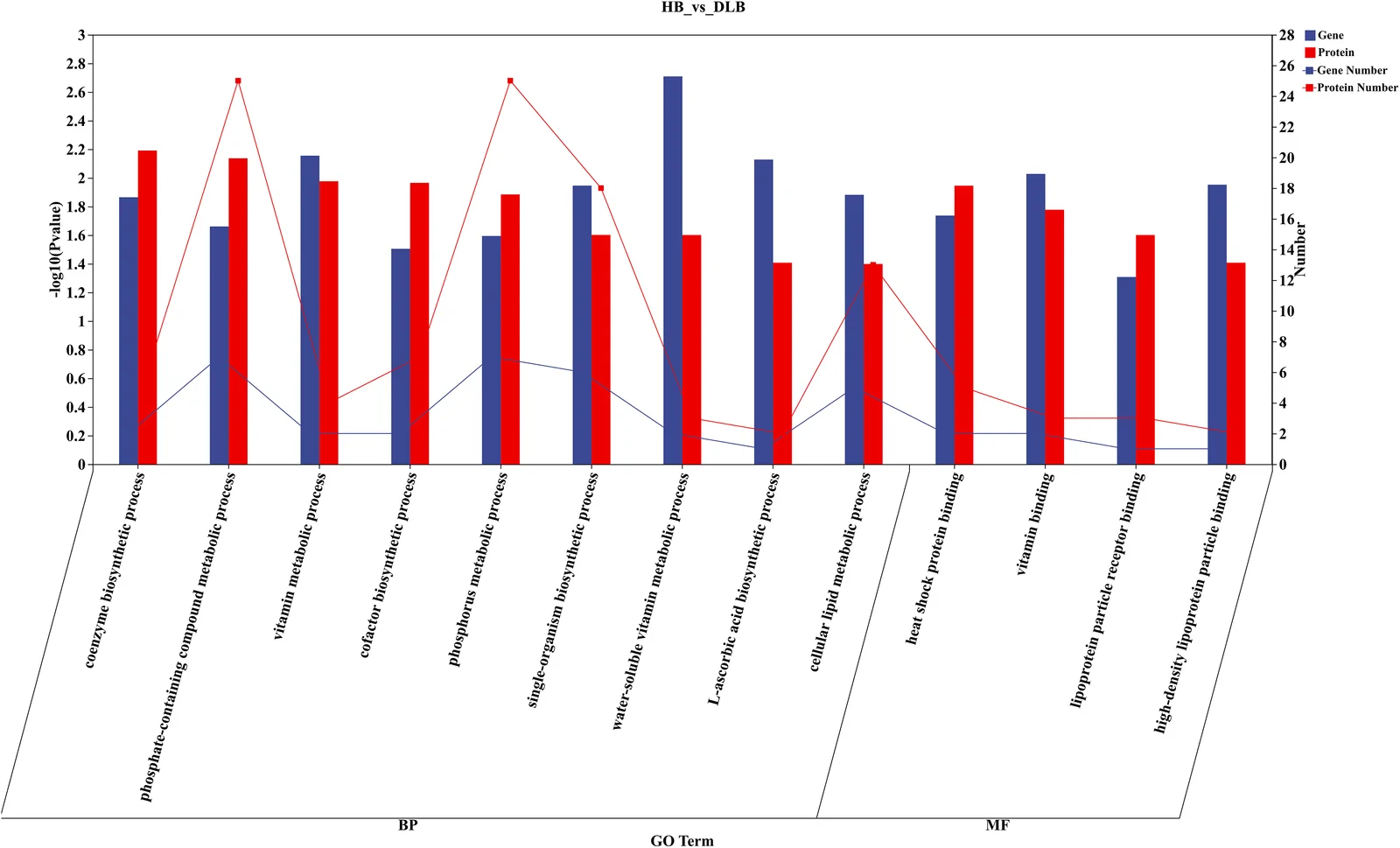
Correlation of GO enrichment between transcriptomic and proteomic datasets.

**Fig 16:**
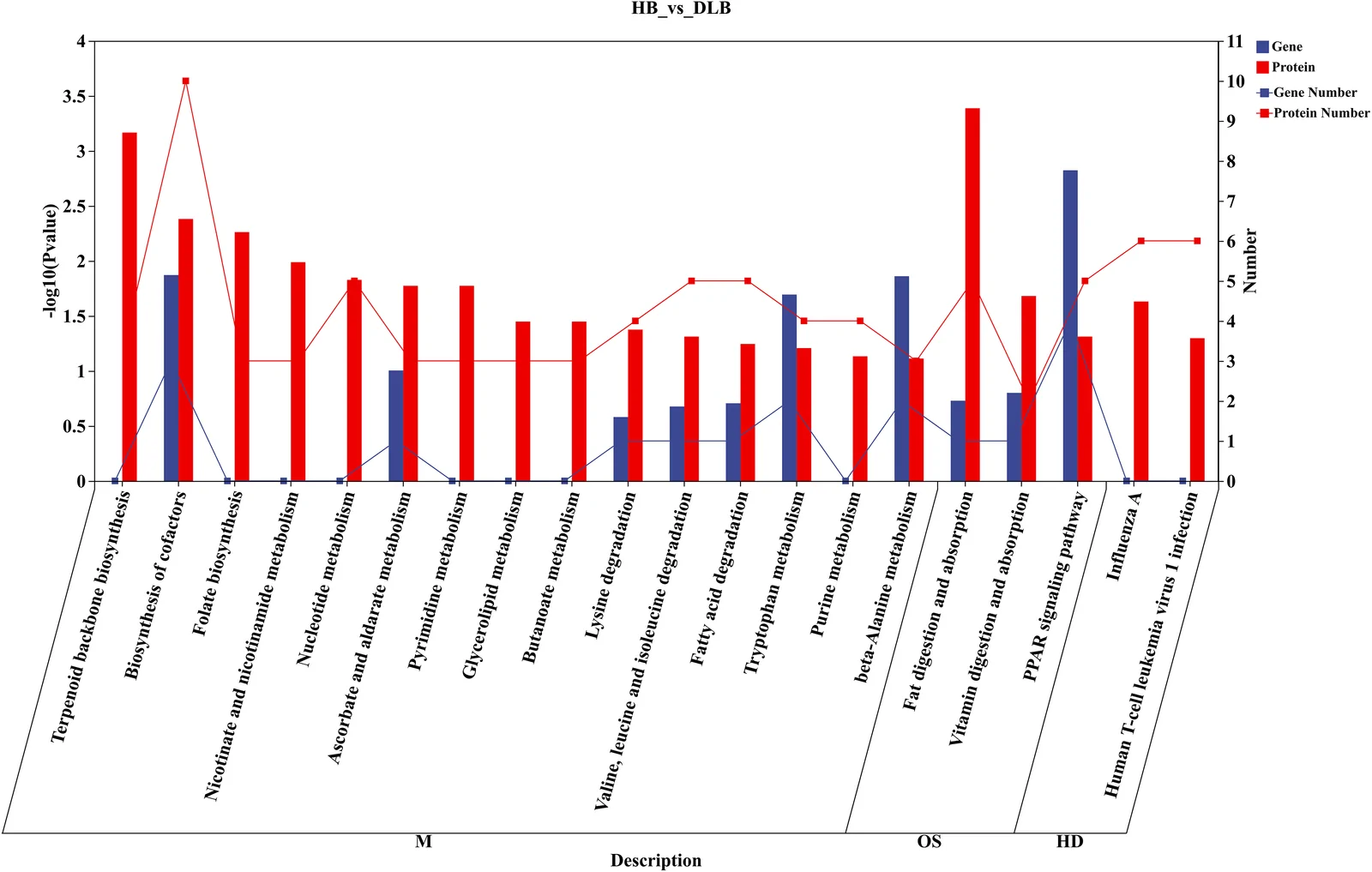
Correlation of KEGG enrichment between transcriptomic and proteomic datasets.

GO enrichment analysis of genes showing consistent expression trends identified significant enrichment in heat shock protein binding, lipoprotein particle receptor binding, and cellular lipid metabolic process (Fig 15). KEGG pathway analysis further revealed that the PPAR signaling pathway, a key regulator of lipid metabolism, was significantly enriched (Fig 16). Key genes involved in this pathway included *CD36*, *SCARB2*, *ACADVL*, *ACADSB*, *ACADM*, *UNC119B*, *PAS4*, *APOA1*, and *PAS-4*.

## Discussions

IMF deposition plays a crucial role in determining meat juiciness, tenderness, and flavor, and thus servesas a key indicator for meat-quality grading. Considerable differences in IMF content exist among pig breeds. Western commercial breeds such as the Duroc pigs, which have undergone for lean meat yield and growth efficiency, typically exhibit low IMF content but high slaughter performance. In contrast, Chinese indigenous breeds such as the Huai pigs are characterized by lower lean percentage and slaughter yield, yet possess significantly higher IMF content and superior sensory meat quality. Despite these well-established phenotypic differences, the molecular mechanisms underlying IMF deposition and meat-quality variation between Chinese and Western pig breeds remain largely unclear.

In this study, proteomic profiling identified 91 DEPs between Huai and Duroc pigs, comprising 34 up-regulated and 57 down-regulated proteins in Huai pigs. KEGG pathway enrichment revealed significant enrichment of several lipid-metabolism- related pathways, including the nicotinate and nicotinamide metabolism, AMPK signaling pathway, cholesterol metabolism, glycerolipid metabolism, and fat digestion and absorption. These pathways are involved in muscle development, energy regulation, and lipid synthesis, all of which directly influence IMF accumulation.

The nicotinate and nicotinamide metabolism pathway plays a key role in lipid homeostasisby generating cofactors essential for fatty-acid oxidation and by interacting with other metabolic cascades [10,11]. Through its modulation of muscle fiber gene expression and fat metabolism, this pathway may regulate muscle fiber type conversion and fiber diameter, ultimately affecting meat texture and flavor [12,13]. Likewise, the AMPK signaling pathway is a central regulator of energy balance and lipid metabolism [14]. As an energy sensor, AMPK detects intracellular energy status and modulates downstream targets controlling protein synthesis, fatty-acid oxidation, and mitochondrial biogenesis [15]. In skeletal muscle an organ central to systemic energy homeostasis-AMPK governs myogenic differentiation, oxidative capacity, and regeneration [16]. When protein synthesis exceeds degradation, AMPK activity supports muscle growth; conversely, excessive activation under energy deficiency promotes proteolysis and atrophy [17]. Notably, KEGG analysis revealed a significant enrichment of the AMPK signaling pathway. As a cellular energy sensor, activated AMPK typically suppresses energy-consuming anabolic processes, including lipid biosynthesis, by down-regulating key lipogenic enzymes such as FASN. In the present study, Huai pigs exhibited a significant up-regulation of FASN, accompanied by substantial IMF deposition. Accordingly, it is speculated that the AMPK signaling pathway is inhibited or maintained at a lower basal activity level in the LD muscle of Huai pigs. This relative attenuation of AMPK activity likely relieves the metabolic inhibition on lipogenesis, thereby facilitating the robust expression of downstream FASN. Consequently, compared to the lean-type Duroc pigs, this persistently active lipid anabolism significantly contributes to the superior intramuscular lipid deposition observed in this indigenous breed.

Genes and proteins involved in adipocyte differentiation, fatty-acid transport, and triglyceride metabolism are critical determinants of IMF deposition. Through PPI analysis and CytoHubba screening, several hub proteins, FASN, EIF4G1, EIF4E, ECHS1, and UPF1, were identified as potential regulators of lipid metabolism and muscle growth. Fatty acid synthase (FASN), a multifunctional enzyme catalyzing the condensation of acetyl-CoA and malonyl-CoA into long-chain fatty acids, plays a pivotal role in de novo lipogenesis and lipid storage [18]. Numerous studies have reported a strong positive correlation between FASN expression and IMF content [19,20]. FASN-derived metabolites can activate the PPARγ signaling pathway, which in turn enhances the expression of adipogenic genes, promoting lipid synthesis and accumulation [21]. Moreover, polymorphisms in FASN have been linked to meat-quality traits, including fatty-acid composition and IMF content [22].

Mitochondrial function also contributes to IMF regulation. Arl2, a regulator of mitochondrial integrity, requires translational activation mediated by DDX60 helicase and EIF4G1 [23,24]. The EIF4G1–EIF4E complex is critical for cap-dependent mRNA translation, influencing protein synthesis and muscle growth [25–27]. Previous studies have shown that miR-322/miR-503 can target EIF4E and EIF4G1, thereby regulating skeletal-muscle differentiation [28]. EIF4E, the primary cap-binding protein, functions downstream of the mTOR signaling pathway, a central node in energy metabolism and lipid synthesis [29,30]. Inhibition of EIF4E decreases SREBP1 expression, a transcription factor crucial for fatty-acid synthesis, suggesting that EIF4E modulates lipid biosynthesis through translational regulation [31]. Collectively, EIF4G1 and EIF4E coordinate protein synthesis and lipid metabolism, influencing IMF composition and deposition [32,33]. ECHS1 (enoyl-CoA hydratase short-chain 1) is a key enzyme in mitochondrial fatty-acid β-oxidation, essential for maintaining energy homeostasis in skeletal muscle [34,35]. Meanwhile, UPF1, an RNA helicase involved in nonsense-mediated mRNA decay (NMD), also functions as an E3 ubiquitin ligase, promoting MYOD degradation and thus modulating myogenic differentiation [36]. Downregulation of UPF1 enhances myogenesis, while its overexpression suppresses it [37]. In the present study, FASN and UPF1 were significantly upregulated in Huai pigs, whereas EIF4G1, EIF4E, and ECHS1 were downregulated relative to Duroc pigs. These findings, further supported by Western blot validation, suggest that Huai pigs exhibit enhanced lipogenic activity and ATP production through upregulated fatty-acid synthesis and oxidation, while concurrently showing reduced protein synthesis, potentially explaining their slower muscle growth but higher IMF deposition.

Furthermore, the integrated analysis revealed a modest correlation between mRNA and protein expression (Pearson’s r = 0.1447), highlighting the prevalence of post-transcriptional and translational regulation during muscle development and intramuscular fat (IMF) deposition. Mechanistically, this uncoupling between transcript and protein abundance can be largely attributed to alterations in both translation efficiency and degradation pathways. As revealed by our proteomic data, the significant down-regulation of the translation initiation factors EIF4G1 and EIF4E in Huai pigs indicates a global or transcript-specific reduction in 5’ cap-dependent translation efficiency. Consequently, elevated mRNA transcripts may not be efficiently translated into functional proteins. Concurrently, the significantly up-regulated UPF1 not only participates in nonsense-mediated mRNA decay but also functions as an E3 ubiquitin ligase to promote protein degradation, adding another layer of dynamic regulation. Therefore, the low mRNA-protein correlation reflects a highly regulated adaptive state in Huai pigs: under this condition, restricted translation efficiency combined with targeted degradation uncouples transcript abundance from actual protein expression levels, thereby favoring IMF deposition over rapid muscle fiber hypertrophy.

To dissect this biological complexity, we stratified the integrative datasets into concordant and discordant candidates. The analysis revealed 576 genes/proteins with concordant expression trends, confirming that for these specific targets, transcriptional regulation still largely aligns with protein-level changes. KEGG analysis of these concordantly expressed genes identified significant enrichment of the PPAR signaling pathway, which is central to lipid metabolism. Key genes involved in this pathway included CD36, SCARB2, ACADVL, ACADSB, ACADM, UNC119B, PAS-4, and APOA1. The PPAR signaling pathway comprises three isoforms-PPARα, PPARβ/δ, and PPARγ-each mediating distinct aspects of lipid metabolism [38]. Among these, PPARγ is predominantly expressed in adipose tissue, where it governs fatty-acid uptake, triglyceride synthesis, and adipocyte differentiation. Activation of PPARγ markedly enhances IMF deposition by upregulating lipogenic genes and suppressing anti-adipogenic factors [39,40]. CD36, a fatty-acid translocase and class B scavenger receptor, facilitates long-chain fatty-acid uptake and regulates lipid transport and metabolism [41,42]. It also influences the expression of ACADVL and ACADM, both key enzymes in β-oxidation, linking fatty-acid uptake with catabolism [43,44]. ACADM, ACADVL, and ACADSB, members of the acyl-CoA dehydrogenase family, catalyze sequential steps in mitochondrial β-oxidation and have been previously associated with IMF metabolism and fatty-acid composition in porcine muscle [45,46].

Taken together, these results indicate that the PPAR signaling pathway promotes fatty-acid uptake and lipid storage, thereby driving substantial intramuscular fat accumulation in Huai pigs compared to Duroc pigs. The coordinated upregulation of lipogenic and β-oxidation-related genes may reflect an adaptive balance between energy production and lipid deposition, contributing to the distinct meat quality traits observed between Chinese and Western pig breeds. Although this study provides profound molecular insights into the differences in IMF deposition mechanisms between Huai and Duroc pigs, we acknowledge certain limitations in the present study (e.g., the relatively limited biological replication). Therefore, the regulatory networks identified herein should be interpreted conservatively as exploratory findings. Future studies incorporating larger sample cohorts and cellular functional experiments are warranted to thoroughly validate these networks and further corroborate the actual biological effects of these key genes and proteins.

## Conclusion

This study provides a comprehensive proteomic and transcriptomic characterization of IMF deposition in Chinese Huai and Western Duroc pigs. Comparative analysis revealed 91 DEPs, primarily enriched in lipid metabolism related pathways, including the AMPK, PPAR, cholesterol metabolism, and fat digestion and absorption pathways. Integration of multi-omics data further identified 576 genes and proteins with concordant expression patterns, among which FASN, EIF4E, EIF4G1, ECHS1, and UPF1 emerged as key regulators of IMF deposition and muscle growth. The results suggest that Huai pigs, compared with Duroc pigs, exhibit enhanced activity of lipid-synthesis and fatty-acid oxidation pathways, contributing to their higher IMF content and superior meat quality. In particular, activation of the PPAR signaling pathway and upregulation of genes such as CD36, ACADVL, ACADSB, and ACADM may accelerate fatty-acid uptake and storage within muscle fibers. Collectively, this study elucidates the molecular mechanisms underlying breed-specific differences in IMF deposition and provides valuable genetic targets and theoretical insights for improving meat quality traits and molecular breeding in pigs.

## Supporting information

S1 File S1 Data. Minimal data set. This file contains the underlying numerical data and statistical values used to generate the figures and charts in this manuscript. **Table S1.** Ranking of candidate key hub proteins in the regulatory network.
(ZIP)
