# Supplementary material for: Integrated analysis of transcriptomic and proteomic profiles identifies divergent regulatory networks for intramuscular fat in Chinese versus Western Pigs
Source: PLoS One. 2026 Sep 18;21(9):e0354756. doi: 10.1371/journal.pone.0354756 (PMC13588535; doi:10.1371/journal.pone.0354756)
Supplement: Supplementary file 1 [file pone.0354756.s001.zip › Supporting Information/Table S1.docx]

| Gene | Degree | Log2FC(HB/DLB) | P-value |
| --- | --- | --- | --- |
| FASN | 5 | 1.1156 | 0.00279 |
| ECHS1 | 4 | -0.4461 | 0.04330 |
| EIF4E | 4 | -0.3548 | 0.03161 |
| EIF4G1 | 4 | -0.3548 | 0.03161 |
| UPF1 | 3 | 1.696 | 0.04412 |
| AGPAT2 | 2 | -1.1225 | 0.04640 |
| AKR1A1 | 2 | -16.6096 | 5.86E-07 |
| APOA1 | 2 | 0.6758 | 0.00790 |
| CBR1 | 2 | 5 | 3.02E-07 |
| EIF3J | 2 | -0.4241 | 0.00188 |
| FDPS | 2 | -1.1172 | 0.03745 |
| NT5C2 | 2 | -0.9473 | 0.02583 |
| PRNP | 2 | -0.8515 | 0.04804 |
| TTR | 2 | 0.9762 | 0.00090 |
| ACAT1 | 1 | 0.7124 | 0.02349 |
| AK3 | 1 | -0.3668 | 0.04594 |
| EEF2K | 1 | -0.6936 | 0.02982 |
| FNTA | 1 | -0.4974 | 0.02629 |
| MMUT | 1 | -1.1162 | 0.01506 |
| NAXE | 1 | 1.2113 | 0.00125 |
| NMNAT1 | 1 | 1.1151 | 0.03850 |
| PDK4 | 1 | -1.5175 | 0.00494 |
| RPS6KA3 | 1 | -0.3838 | 0.04914 |
| RUVBL1 | 1 | -0.5754 | 0.02315 |

**Table S1. Ranking of candidate key hub proteins in the regulatory network.**
