# Supplementary material for: Integrated analysis of transcriptomic and proteomic profiles identifies divergent regulatory networks for intramuscular fat in Chinese versus Western Pigs
Source: PLoS One. 2026 Sep 18;21(9):e0354756. doi: 10.1371/journal.pone.0354756 (PMC13588535; doi:10.1371/journal.pone.0354756)
Supplement: Supplementary file 2 [file pone.0354756.s003.doc]

Dear Dr. Wang,

We've checked your submission and before we can proceed, we need you to address the following issues:

1.Please upload a Response to Reviewers letter which should include a point by point response to each of the points made by the Editor and / or Reviewers. (This should be uploaded as a 'Response to Reviewers' file type.) Please follow this link for more information: http://blogs.PLOS.org/everyone/2011/05/10/how-to-submit-your-revised-manuscript/
Reply: We thank the Editor for the detailed instructions. We have carefully prepared this point-by-point Response to Reviewers letter, addressing all comments and suggestions raised by both the Editor and the Reviewers. We confirm that this document has been successfully uploaded into the submission system under the required 'Response to Reviewers' file type.
2.Please note that PLOS ONE requires submissions reporting blot or gel data to comply in full with the reporting requirements described at https://journals.plos.org/plosone/s/figures#loc-blot-and-gel-reporting-requirements. We now require authors to provide the original unadjusted and uncropped images for any blot or gel data reported in PLOS ONE submissions. In our internal checks for your submission, we noted that you did not provide original raw image files supporting blot/gel data in response to our previous request.
Please provide the unadjusted and uncropped images underlying all blot and gel figures at this time; see the above URL for instructions on how the raw blot/gel image data should be prepared and submitted. Note that we cannot consider your manuscript further until you have met this requirement. If there are any blot/gel figures in your manuscript or its Supporting Information for which you cannot provide the original uncropped and unadjusted images please specify this in your response and provide details as to which figures are affected by raw image data unavailability. Email us at plosone@plos.org if you have questions.
Reply: We sincerely apologize for the oversight of omitting the original, uncropped blot images in our previous submission. To fully comply with the journal's guidelines, we have now carefully prepared and uploaded a separate supplementary file named S1_Raw_Images.pdf. This file contains the original, unadjusted, and uncropped full-membrane image files for all Western blot data reported in this study. We hope this complete dataset satisfies the journal's data transparency requirements.

3.We notice that your revision was submitted on March 30 2026, but the manuscript file in your submission's file inventory was uploaded on Jan 05 2026. Please upload the latest version of your revised manuscript as the main article file, with the item type 'Manuscript,' ensuring that it does not contain any tracked changes or highlighting. This will be used in the production process if your manuscript is accepted.
Reply: We sincerely apologize for the file upload error during our previous submission process.

We have now corrected this issue. The latest, fully updated version of our revised manuscript has been successfully uploaded. Per your instructions, this file has been categorized under the item type 'Manuscript' and is a clean version, containing absolutely no tracked changes, colored text, or highlighting. We confirm that this final version is ready for the production process should our manuscript be accepted.

4.Thank you for updating your data availability statement. You note that your data are available within the Supporting Information files, but no such files have been included with your submission. At this time we ask that you please upload your minimal data set as a Supporting Information file, or to a public repository such as Figshare or Dryad.
Please also ensure that when you upload your file you include separate captions for your supplementary files at the end of your manuscript.
As soon as you confirm the location of the data underlying your findings, we will be able to proceed with the review of your submission.
Reply: We sincerely apologize for any confusion regarding our data availability statement. First, we would like to clarify that all original raw sequencing data (transcriptome and proteomics) have already been publicly deposited in the appropriate databases. The transcriptome data is available at the NCBI database under the accession number PRJNA962316, and the proteomics data is available at ProteomeXchange under the identifier PXD072414. We have updated our Data Availability Statement in the manuscript to reflect this explicitly. Second, to fully comply with the journal's policy regarding the "minimal data set" underlying our findings, we have now prepared and uploaded a separate Supporting Information file (S1_Data.xlsx). This file contains all the underlying numerical values and statistical data used to generate the charts and graphs in this study (e.g., the individual data points for Figures 10, 11, 12, etc.). Finally, as requested, we have ensured that separate captions for all supplementary files (including Table S1 and S1_Data.xlsx) are clearly listed at the very end of the revised manuscript.
5.Please ensure that you refer to Figure 16 in your text as, if accepted, production will need this reference to link the reader to the figure.
Reply: We sincerely apologize for this oversight. We have carefully reviewed the manuscript and ensured that Figure 16 is now explicitly cited in the relevant section of the main text. This will ensure proper linkage during the production process.
6.Please upload a copy of Figure 17 which you refer to in your text. Or if the figure is no longer to be included as part of the submission please remove all reference to it within the text.
Reply: We sincerely apologize for the confusion. We confirm that there is no Figure 17 in our study. The mention of "Figure 17" in the previous version of the manuscript was merely a typographical numbering error. We have now carefully reviewed the text and corrected/removed all erroneous references to Figure 17 in the revised manuscript.
7.Please include a separate caption for each figure 16 in your manuscript.

Reply: We apologize for this omission. We have now added the missing separate caption for Figure 16 in the revised manuscript. All figure captions are now completely and clearly listed according to the journal's guidelines.
