# Supplementary material for: Integrated analysis of transcriptomic and proteomic profiles identifies divergent regulatory networks for intramuscular fat in Chinese versus Western Pigs
Source: PLoS One. 2026 Sep 18;21(9):e0354756. doi: 10.1371/journal.pone.0354756 (PMC13588535; doi:10.1371/journal.pone.0354756)
Supplement: Peer review history

# Peer review: Decision Letter 0

**DOI:** 10.1371/journal.pone.0354756.r001

**Authors:** Mohammad Moniruzzaman

30 Mar 2026

Dear Dr. Wang,

Thank you for submitting your manuscript to PLOS ONE. After careful consideration, we feel that it has merit but does not fully meet PLOS ONE’s publication criteria as it currently stands. Therefore, we invite you to submit a revised version of the manuscript that addresses the points raised during the review process.

Please submit your revised manuscript by May 14 2026 11:59PM. If you will need more time than this to complete your revisions, please reply to this message or contact the journal office at plosone@plos.org. When you're ready to submit your revision, log on to https://www.editorialmanager.com/pone/ and select the 'Submissions Needing Revision' folder to locate your manuscript file.

- A letter that responds to each point raised by the academic editor and reviewer(s). You should upload this letter as a separate file labeled 'Response to Reviewers'.
- A marked-up copy of your manuscript that highlights changes made to the original version. You should upload this as a separate file labeled 'Revised Manuscript with Track Changes'.
- An unmarked version of your revised paper without tracked changes. You should upload this as a separate file labeled 'Manuscript'.

If you would like to make changes to your financial disclosure, please include your updated statement in your cover letter. Guidelines for resubmitting your figure files are available below the reviewer comments at the end of this letter.

If applicable, we recommend that you deposit your laboratory protocols in protocols.io to enhance the reproducibility of your results. Protocols.io assigns your protocol its own identifier (DOI) so that it can be cited independently in the future. For instructions see: https://journals.plos.org/plosone/s/submission-guidelines#loc-laboratory-protocols. Additionally, PLOS ONE offers an option for publishing peer-reviewed Lab Protocol articles, which describe protocols hosted on protocols.io. Read more information on sharing protocols at https://plos.org/protocols?utm_medium=editorial-email&utm_source=authorletters&utm_campaign=protocols.

As the corresponding author, your ORCID iD is verified in the submission system and will appear in the published article. PLOS supports the use of ORCID, and we encourage all coauthors to register for an ORCID iD and use it as well. Please encourage your coauthors to verify their ORCID iD within the submission system before final acceptance, as unverified ORCID iDs will not appear in the published article. *Only* the individual author can complete the verification step; PLOS staff *cannot* verify ORCID iDs on behalf of authors.

We look forward to receiving your revised manuscript.

Kind regards,

Mohammad Moniruzzaman

Academic Editor

PLOS One

Journal requirements:

When submitting your revision, we need you to address these additional requirements.

1. Please ensure that your manuscript meets PLOS ONE's style requirements, including those for file naming. The PLOS ONE style templates can be found at

https://journals.plos.org/plosone/s/file?id=wjVg/PLOSOne_formatting_sample_main_body.pdf and

https://journals.plos.org/plosone/s/file?id=ba62/PLOSOne_formatting_sample_title_authors_affiliations.pdf

2. Please note that PLOS One has specific guidelines on code sharing for submissions in which author-generated code underpins the findings in the manuscript. In these cases, we expect all author-generated code to be made available without restrictions upon publication of the work. Please review our guidelines at https://journals.plos.org/plosone/s/materials-and-software-sharing#loc-sharing-code and ensure that your code is shared in a way that follows best practice and facilitates reproducibility and reuse.

3. PLOS ONE now requires that authors provide the original uncropped and unadjusted images underlying all blot or gel results reported in a submission’s figures or Supporting Information files. This policy and the journal’s other requirements for blot/gel reporting and figure preparation are described in detail at https://journals.plos.org/plosone/s/figures#loc-blot-and-gel-reporting-requirements and https://journals.plos.org/plosone/s/figures#loc-preparing-figures-from-image-files. When you submit your revised manuscript, please ensure that your figures adhere fully to these guidelines and provide the original underlying images for all blot or gel data reported in your submission. See the following link for instructions on providing the original image data: https://journals.plos.org/plosone/s/figures#loc-original-images-for-blots-and-gels.

In your cover letter, please note whether your blot/gel image data are in Supporting Information or posted at a public data repository, provide the repository URL if relevant, and provide specific details as to which raw blot/gel images, if any, are not available. Email us at plosone@plos.org if you have any questions.

If the reviewer comments include a recommendation to cite specific previously published works, please review and evaluate these publications to determine whether they are relevant and should be cited. There is no requirement to cite these works unless the editor has indicated otherwise.

Please review your reference list to ensure that it is complete and correct. If you have cited papers that have been retracted, please include the rationale for doing so in the manuscript text, or remove these references and replace them with relevant current references. Any changes to the reference list should be mentioned in the rebuttal letter that accompanies your revised manuscript. If you need to cite a retracted article, indicate the article’s retracted status in the References list and also include a citation and full reference for the retraction notice.

[Note: HTML markup is below. Please do not edit.]

Reviewers' comments:

Reviewer's Responses to Questions

**Comments to the Author**

1. Is the manuscript technically sound, and do the data support the conclusions?

Reviewer #1: Yes

Reviewer #2: Yes

**********

2. Has the statistical analysis been performed appropriately and rigorously?

Reviewer #1: Yes

Reviewer #2: Yes

**********

3. Have the authors made all data underlying the findings in their manuscript fully available?

The PLOS Data policy

Reviewer #1: Yes

Reviewer #2: Yes

**********

4. Is the manuscript presented in an intelligible fashion and written in standard English?

Reviewer #1: Yes

Reviewer #2: Yes

**********

Reviewer #1: Review Comments on Scientific Research Papers

Reference#: PLOS-One-2026-226

Title: Integrated Analysis of Transcriptomic and Proteomic Profiles Identifies Divergent

Regulatory Networks for Intramuscular Fat in Chinese versus Western Pigs

This manuscript addresses a breeding-relevant question by comparing fat deposition–related molecular differences between an indigenous Chinese pig and a Western lean breed using multi-omics data from longissimus dorsi muscle. The topic is within the scope of animal breeding research, and the overall structure is clear. The analyses are generally coherent and the manuscript has potential value as a resource for hypothesis generation.

In my opinion, the study can be considered for acceptance after minor revision. To improve clarity, rigor, and avoid over-interpretation, I suggest the authors revise the following points:

(1) Key animal information is incomplete. Because fat deposition dynamics can differ between indigenous and Western breeds, and can vary strongly by developmental stage, the authors should explicitly report basic metadata (at minimum: age/day-of-life at slaughter, body weight, castration timing, diet/feeding regime, and batch). Without these details, it is difficult to interpret the results as “IMF deposition mechanisms” rather than breed differences at an unspecified stage.

(2) The biological replication is limited (n=3 per breed). Please add clearer sample-level quality control and robustness presentation (e.g., PCA/clustering, sample–sample correlation, outlier assessment, and individual-level expression/abundance plots for key features). In addition, conclusions should be phrased conservatively in the Discussion to reflect the small sample size.

(3) Western blot validation of only three targets provides limited support for pathway/network-level conclusions. Please clarify the rationale for selecting these three proteins and, where possible, prioritize validation of proteins that are central to the main mechanistic claims (e.g., key nodes/hub candidates in the reported network or core pathways). If reagent availability is a constraint, the limitation should be explicitly stated, and a supplementary table ranking candidate targets (effect size, FDR, pathway membership/centrality) would be helpful.

(4) The manuscript reports low overall concordance between transcript and protein levels. This observation may have multiple explanations (biological and technical), therefore it should not be used as direct evidence for post-transcriptional regulation without additional supporting analyses or experiments. Please revise the wording accordingly and consider presenting results stratified by concordant vs. discordant candidates.

Reviewer #2: This manuscript investigates the molecular mechanisms underlying the differences in intramuscular fat (IMF) deposition between the Chinese indigenous Huai pig and the Western Duroc pig through an integrated DIA proteomic and transcriptomic analysis. The overall rationale is clear, but there is still room for improvement regarding methodological details, data interpretation, and figure formatting.

1.Line 153 and 216: When screening differentially expressed proteins (DEPs) and performing GO and KEGG enrichment analyses, the threshold was set to p < 0.05. Were these p-values subjected to multiple testing correction (e.g., Benjamini-Hochberg FDR correction)? If not, the risk of false positives is high; if so, please specify the correction method used in the statistical analysis part of the "Materials and Methods" section.

2.Line 229: KEGG analysis revealed significant enrichment of the AMPK signaling pathway. Typically, AMPK activation suppresses lipid synthesis (e.g., by inhibiting FASN). Given that FASN was significantly upregulated and IMF deposition is higher in Huai pigs, please explicitly state in the discussion whether the AMPK pathway is activated or inhibited in the longissimus dorsi muscle of Huai pigs. How does this logically align with the high expression of downstream FASN?

3.Line 262: The data showed that the overall correlation between mRNA and protein expression was modest (Pearson's r = 0.1447). Although post-transcriptional regulation was mentioned, it is recommended to explore the specific biological mechanisms further in the Discussion section: during muscle tissue development or lipid deposition, is this low correlation caused by changes in translation efficiency or differences in protein degradation rates?

Line 358: In the combined bar and line charts for Figure 14 (GO enrichment) and Figure 15 (KEGG enrichment), the font for the technical term labels on the X-axis is too small and crowded, making it extremely difficult to read. Please adjust the layout of the charts and enlarge the font size to ensure clarity for printing and reading.

**********

what does this mean?). If published, this will include your full peer review and any attached files.

If you choose “no”, your identity will remain anonymous but your review may still be made public.

**Do you want your identity to be public for this peer review?** For information about this choice, including consent withdrawal, please see our Privacy Policy

Reviewer #1: **Yes:**

Reviewer #2: **Yes:**

**********

[NOTE: If reviewer comments were submitted as an attachment file, they will be attached to this email and accessible via the submission site. Please log into your account, locate the manuscript record, and check for the action link "View Attachments". If this link does not appear, there are no attachment files.]

To ensure your figures meet our technical requirements, please review our figure guidelines: https://journals.plos.org/plosone/s/figures

You may also use PLOS’s free figure tool, NAAS, to help you prepare publication quality figures: https://journals.plos.org/plosone/s/figures#loc-tools-for-figure-preparation.

NAAS will assess whether your figures meet our technical requirements by comparing each figure against our figure specifications.

# Author comment: Author response to Decision Letter 1

**DOI:** 10.1371/journal.pone.0354756.r002

1 Jun 2026

Reviewer #1: Review Comments on Scientific Research Papers

Reference#: PLOS-One-2026-226

Title: Integrated Analysis of Transcriptomic and Proteomic Profiles Identifies Divergent Regulatory Networks for Intramuscular Fat in Chinese versus Western Pigs

This manuscript addresses a breeding-relevant question by comparing fat deposition–related molecular differences between an indigenous Chinese pig and a Western lean breed using multi-omics data from longissimus dorsi muscle. The topic is within the scope of animal breeding research, and the overall structure is clear. The analyses are generally coherent and the manuscript has potential value as a resource for hypothesis generation.

In my opinion, the study can be considered for acceptance after minor revision. To improve clarity, rigor, and avoid over-interpretation, I suggest the authors revise the following points:

(1) Key animal information is incomplete. Because fat deposition dynamics can differ between indigenous and Western breeds, and can vary strongly by developmental stage, the authors should explicitly report basic metadata (at minimum: age/day-of-life at slaughter, body weight, castration timing, diet/feeding regime, and batch). Without these details, it is difficult to interpret the results as “IMF deposition mechanisms” rather than breed differences at an unspecified stage.

Reply: We are very grateful for the reviewer's constructive comment. Accordingly, we have comprehensively updated the Materials and Methods section in the revised manuscript to include detailed information regarding castration timing, exact slaughter age, body weights, and standardized electrical stunning procedures. All relevant modifications have been highlighted in red (Lines 112-117).

(2) The biological replication is limited (n=3 per breed). Please add clearer sample-level quality control and robustness presentation (e.g., PCA/clustering, sample–sample correlation, outlier assessment, and individual-level expression/abundance plots for key features). In addition, conclusions should be phrased conservatively in the Discussion to reflect the small sample size.

Reply:We sincerely appreciate the reviewer’s rigorous evaluation of our data. We completely agree that a biological replication of n=3 per breed is relatively limited, which necessitates highly transparent quality control and cautious interpretation of the results. First, we have incorporated a sample-to-sample Pearson correlation heatmap with hierarchical clustering into the Results section (Fig. 4). This visualization demonstrates excellent intra-group consistency (Pearson’s R > 0.95) and clear inter-group separation, effectively ruling out the presence of extreme outliers driving the group differences. Second, following your suggestion, for the key proteins validated by Western blot (PDK4, EIF4E, and ACAT1), we have supplemented their proteomic sequencing expression bar charts (Fig. 13) （Line 257-259）, explicitly displaying the individual-level data points (scatter dots) overlaid on the bars. This provides full transparency regarding the biological variance and individual sample distributions within our small cohort. Finally, we have carefully refined the phrasing throughout the Discussion section. We also added a dedicated paragraph addressing the limitations of the sample size, framing our multi-omics findings as exploratory models that warrant future large-scale validations. All modifications have been highlighted in red in the revised manuscript (Line 398-404).

(3) Western blot validation of only three targets provides limited support for pathway/network-level conclusions. Please clarify the rationale for selecting these three proteins and, where possible, prioritize validation of proteins that are central to the main mechanistic claims (e.g., key nodes/hub candidates in the reported network or core pathways). If reagent availability is a constraint, the limitation should be explicitly stated, and a supplementary table ranking candidate targets (effect size, FDR, pathway membership/centrality) would be helpful.

Reply: We sincerely thank the reviewer for this highly constructive comment and for considering our practical difficulties. We completely agree that validating only three targets provides limited support for complex network-level conclusions, and we deeply appreciate your understanding of the objective constraints regarding reagent and antibody availability for non-model organisms (pigs). To clarify the rationale, we selected ACAT1, PDK4, and EIF4E for Western blot validation precisely because they are critical representative nodes bridging the core metabolic and translational pathways reported in this study. For instance, ACAT1 and PDK4 are key metabolic enzymes controlling lipid and energy homeostasis, while EIF4E acts as a central hub (Degree = 4) in the translation initiation network. We initially considered other top-ranked hub proteins (such as FASN and UPF1), but unfortunately, they failed to yield specific bands during the optimization of available commercial antibodies. Following your excellent suggestion, we have added a new supplementary table (Table S1). This table ranks the core candidate targets (including FASN, UPF1, etc.) that urgently require future validation based on their effect size (Log2FC), statistical significance, and pathway centrality (Degree in the PPI network) (Line 243).

(4) The manuscript reports low overall concordance between transcript and protein levels. This observation may have multiple explanations (biological and technical), therefore it should not be used as direct evidence for post-transcriptional regulation without additional supporting analyses or experiments. Please revise the wording accordingly and consider presenting results stratified by concordant vs. discordant candidates.

Reply: We sincerely thank the reviewer for this highly insightful and constructive comment. We completely agree that the overall low concordance between transcript and protein levels can arise from a combination of biological factors and technical/temporal factors (differing detection sensitivities between RNA-seq and LC-MS/MS, and the inherent temporal delay between mRNA transcription and protein synthesis). Therefore, we have carefully revised the wording in the manuscript to clarify that low correlation alone is not "direct evidence" of post-transcriptional regulation. However, to provide the "additional supporting analyses" you recommended, we further investigated the translational and degradation machinery in our datasets. Our proteomic results revealed a significant down-regulation of key translation initiation factors (EIF4G1 and EIF4E) in Huai pigs, indicating a global or transcript-specific reduction in 5' cap-dependent translation efficiency. Concurrently, the significantly up-regulated UPF1 not only participates in nonsense-mediated mRNA decay but also functions as an E3 ubiquitin ligase to promote protein degradation. Taken together, this restricted translation efficiency combined with targeted degradation provides solid biological evidence for the uncoupling of transcript abundance from actual protein expression levels, which favors IMF deposition over rapid muscle fiber hypertrophy.

Finally, following your valuable suggestion, we have stratified our multi-omics results into concordant (synchronized changes in both levels) and discordant (opposite trends or significant changes in only one omics layer) candidates. These stratified results are now presented in Figure 13. We have added this comprehensive discussion to the revised manuscript and marked all changes in red (Line 262-271 and Line 358-376 ).

Reviewer #2: This manuscript investigates the molecular mechanisms underlying the differences in intramuscular fat (IMF) deposition between the Chinese indigenous Huai pig and the Western Duroc pig through an integrated DIA proteomic and transcriptomic analysis. The overall rationale is clear, but there is still room for improvement regarding methodological details, data interpretation, and figure formatting.

1.Line 153 and 216: When screening differentially expressed proteins (DEPs) and performing GO and KEGG enrichment analyses, the threshold was set to p < 0.05. Were these p-values subjected to multiple testing correction (e.g., Benjamini- Hochberg FDR correction)? If not, the risk of false positives is high; if so, please specify the correction method used in the statistical analysis part of the "Materials and Methods" section.

Reply：Thank you for the valuable comments from the reviewer. The corrections have been made in the newly submitted paper and are marked in red. Thank you for your meticulous review.

2.Line 229: KEGG analysis revealed significant enrichment of the AMPK signaling pathway. Typically, AMPK activation suppresses lipid synthesis (e.g., by inhibiting FASN). Given that FASN was significantly upregulated and IMF deposition is higher in Huai pigs, please explicitly state in the discussion whether the AMPK pathway is activated or inhibited in the longissimus dorsi muscle of Huai pigs. How does this logically align with the high expression of downstream FASN?

Reply: We sincerely thank the reviewer for this insightful and highly professional comment. As a cellular energy sensor, activated AMPK typically suppresses energy-consuming anabolic processes (including lipid biosynthesis) by down-regulating key lipogenic enzymes such as FASN. Given our findings of significant FASN up-regulation and substantial IMF deposition in Huai pigs, this indicates that, compared to Duroc pigs, the AMPK signaling pathway in the longissimus dorsi muscle of Huai pigs is actually in an inhibited state. This relative inhibition of AMPK activity effectively relieves the metabolic inhibition on lipogenesis, thereby permitting the robust expression of downstream FASN and further enhancing IMF deposition. We have substantially revised the Discussion section accordingly, and the relevant modifications have been marked in red in the revised manuscript.

3.Line 262: The data showed that the overall correlation between mRNA and protein expression was modest (Pearson's r =0.1447). Although post-transcriptional regulation was mentioned, it is recommended to explore the specific biological mechanisms further in the Discussion section: during muscle tissue development or lipid deposition, is this low correlation caused by changes in translation efficiency or differences in protein degradation rates?

Reply: We sincerely thank the reviewer for this highly insightful and constructive comment. Our proteomic results revealed a significant down-regulation of key translation initiation factors (EIF4G1 and EIF4E) in Huai pigs, indicating a global or transcript-specific reduction in 5' cap-dependent translation efficiency. Consequently, elevated mRNA transcripts may not be efficiently translated into functional proteins. Concurrently, the significantly up-regulated UPF1 not only participates in nonsense-mediated mRNA decay but also functions as an E3 ubiquitin ligase to promote protein degradation, thereby adding another layer of dynamic regulation. Taken together, restricted translation efficiency combined with targeted degradation uncouples transcript abundance from actual protein expression levels, which favors IMF deposition over rapid muscle fiber hypertrophy. We have added this detailed mechanistic discussion to the Discussion section of the main text and marked the changes in red.

4. Line 358: In the combined bar and line charts for Figure 14 (GO enrichment) and Figure 15 (KEGG enrichment), the font for the technical term labels on the X-axis is too small and crowded, making it extremely difficult to read. Please adjust the layout of the charts and enlarge the font size to ensure clarity for printing and reading.

Reply: We sincerely thank the reviewer for pointing out this issue. We have carefully replotted Figure 14 and Figure 15. The overall layout of the charts has been optimized, and the font size of the X-axis labels has been significantly enlarged to prevent text crowding. The updated high-resolution figures have been inserted into the revised manuscript.

Dear Dr. Wang,

We've checked your submission and before we can proceed, we need you to address the following issues:

1.Please upload a Response to Reviewers letter which should include a point by point response to each of the points made by the Editor and / or Reviewers. (This should be uploaded as a 'Response to Reviewers' file type.) Please follow this link for more information: http://blogs.PLOS.org/everyone/2011/05/10/how-to-submit-your-revised-manuscript/

Reply: We thank the Editor for the detailed instructions. We have carefully prepared this point-by-point Response to Reviewers letter, addressing all comments and suggestions raised by both the Editor and the Reviewers. We confirm that this document has been successfully uploaded into the submission system under the required 'Response to Reviewers' file type.

2.Please note that PLOS ONE requires submissions reporting blot or gel data to comply in full with the reporting requirements described at https://journals.plos.org/plosone/s/figures#loc-blot-and-gel-reporting-requirements. We now require authors to provide the original unadjusted and uncropped images for any blot or gel data reported in PLOS ONE submissions. In our internal checks for your submission, we noted that you did not provide original raw image files supporting blot/gel data in response to our previous request.

Please provide the unadjusted and uncropped images underlying all blot and gel figures at this time; see the above URL for instructions on how the raw blot/gel image data should be prepared and submitted. Note that we cannot consider your manuscript further until you have met this requirement. If there are any blot/gel figures in your manuscript or its Supporting Information for which you cannot provide the original uncropped and unadjusted images please specify this in your response and provide details as to which figures are affected by raw image data unavailability. Email us at plosone@plos.org if you have questions.

Reply: We sincerely apologize for the oversight of omitting the original, uncropped blot images in our previous submission. To fully comply with the journal's guidelines, we have now carefully prepared and uploaded a separate supplementary file named S1_Raw_Images.pdf. This file contains the original, unadjusted, and uncropped full-membrane image files for all Western blot data reported in this study. We hope this complete dataset satisfies the journal's data transparency requirements.

3.We notice that your revision was submitted on March 30 2026, but the manuscript file in your submission's file inventory was uploaded on Jan 05 2026. Please upload the latest version of your revised manuscript as the main article file, with the item type 'Manuscript,' ensuring that it does not contain any tracked changes or highlighting. This will be used in the production process if your manuscript is accepted.

Reply: We sincerely apologize for the file upload error during our previous submission process.

We have now corrected this issue. The latest, fully updated version of our revised manuscript has been successfully uploaded. Per your instructions, this file has been categorized under the item type 'Manuscript' and is a clean version, containing absolutely no tracked changes, colored text, or highlighting. We confirm that this final version is ready for the production process should our manuscript be accepted.

4.Thank you for updating your data availability statement. You note that your data are available within the Supporting Information files, but no such files have been included with your submission. At this time we ask that you please upload your minimal data set as a Supporting Information file, or to a public repository such as Figshare or Dryad.

Please also ensure that when you upload your file you include separate captions for your supplementary files at the end of your manuscript.

As soon as you confirm the location of the data underlying your findings, we will be able to proceed with the review of your submission.

Reply: We sincerely apologize for any confusion regarding our data availability statement. First, w

Attachment Submitted filename: Editor.doc

# Peer review: Decision Letter 1

**DOI:** 10.1371/journal.pone.0354756.r003

**Authors:** Mohammad Moniruzzaman

12 Jul 2026

<p>Integrated Analysis of Transcriptomic and Proteomic Profiles Identifies Divergent Regulatory Networks for Intramuscular Fat in Chinese versus Western Pigs

PONE-D-26-00768R1

Dear Dr. Wang,

We’re pleased to inform you that your manuscript has been judged scientifically suitable for publication and will be formally accepted for publication once it meets all outstanding technical requirements.

Within one week, you’ll receive an e-mail detailing the required amendments. When these have been addressed, you’ll receive a formal acceptance letter and your manuscript will be scheduled for publication.

An invoice will be generated when your article is formally accepted. Please note, if your institution has a publishing partnership with PLOS and your article meets the relevant criteria, all or part of your publication costs will be covered. Please make sure your user information is up-to-date by logging into Editorial Manager at Editorial Manager® and clicking the ‘Update My Information' link at the top of the page. For questions related to billing, please contact billing support.

If your institution or institutions have a press office, please notify them about your upcoming paper to help maximize its impact. If they’ll be preparing press materials, please inform our press team as soon as possible -- no later than 48 hours after receiving the formal acceptance. Your manuscript will remain under strict press embargo until 2 pm Eastern Time on the date of publication. For more information, please contact onepress@plos.org.

Kind regards,

Mohammad Moniruzzaman

Academic Editor

PLOS One

Additional Editor Comments (optional):

Reviewers' comments:

Reviewer's Responses to Questions

**Comments to the Author**

Reviewer #1: All comments have been addressed

Reviewer #2: (No Response)

**********

2. Is the manuscript technically sound, and do the data support the conclusions?

Reviewer #1: Yes

Reviewer #2: (No Response)

**********

3. Has the statistical analysis been performed appropriately and rigorously?

Reviewer #1: Yes

Reviewer #2: (No Response)

**********

4. Have the authors made all data underlying the findings in their manuscript fully available?

The PLOS Data policy

Reviewer #1: Yes

Reviewer #2: (No Response)

**********

5. Is the manuscript presented in an intelligible fashion and written in standard English?

Reviewer #1: Yes

Reviewer #2: (No Response)

**********

Reviewer #1: Dear Author,

After careful review, I found that all the issues of concern to the reviewers have been perfectly resolved. At the same time, I suggest the author consider adding a table to display the potential associations between the IMF related candidate genes identified in the article and their chromosomal loci, biological functions, and IMF.

I strongly recommend that the editor consider accepting the publication of the paper as soon as possible.

Reviewer #2: (No Response)

**********

what does this mean?). If published, this will include your full peer review and any attached files.

If you choose “no”, your identity will remain anonymous but your review may still be made public.

**Do you want your identity to be public for this peer review?** For information about this choice, including consent withdrawal, please see our Privacy Policy

Reviewer #1: **Yes:** Xiangxing Zhu

Reviewer #2: No

**********

# Editor report: Acceptance letter

**DOI:** 10.1371/journal.pone.0354756.r004

**Authors:** Mohammad Moniruzzaman

PONE-D-26-00768R1

PLOS One

Dear Dr. Wang,

I'm pleased to inform you that your manuscript has been deemed suitable for publication in PLOS One. Congratulations! Your manuscript is now being handed over to our production team.

At this stage, our production department will prepare your paper for publication. This includes ensuring the following:

* All references, tables, and figures are properly cited

* All relevant supporting information is included in the manuscript submission,

* There are no issues that prevent the paper from being properly typeset

You will receive further instructions from the production team, including instructions on how to review your proof when it is ready. Please keep in mind that we are working through a large volume of accepted articles, so please give us a few days to review your paper and let you know the next and final steps.

Lastly, if your institution or institutions have a press office, please let them know about your upcoming paper now to help maximize its impact. If they'll be preparing press materials, please inform our press team within the next 48 hours. Your manuscript will remain under strict press embargo until 2 pm Eastern Time on the date of publication. For more information, please contact onepress@plos.org.

You will receive an invoice from PLOS for your publication fee after your manuscript has reached the completed accept phase. If you receive an email requesting payment before acceptance or for any other service, this may be a phishing scheme. Learn how to identify phishing emails and protect your accounts at https://explore.plos.org/phishing.

If we can help with anything else, please email us at customercare@plos.org.

Thank you for submitting your work to PLOS One and supporting open access.

Kind regards,

PLOS One Editorial Office Staff

on behalf of

Dr. Mohammad Moniruzzaman

Academic Editor

PLOS One
